# NO_2_ Adsorption on Oxygen-Modified Ag at Ambient Conditions

**DOI:** 10.1021/jacs.5c16683

**Published:** 2025-11-05

**Authors:** Alvaro Posada-Borbón, Trenton Wolter, Huaizhe Yu, Evangelos Smith, James J. Schauer, Reid C. Van Lehn, Victor M. Zavala, Nicholas L. Abbott, Manos Mavrikakis

**Affiliations:** † Department of Chemical and Biological Engineering, 5228 University of Wisconsin-Madison, Madison, Wisconsin 53706, United States; ‡ Robert Frederick Smith School of Chemical and Biomolecular Engineering, 5922 Cornell University, 1 Ho Plaza, Ithaca, New York 14853, United States

## Abstract

Silver-based materials are promising for the removal and detection of NO_2_ via surface reactions. Under ambient conditions, NO_2_ has been reported to adsorb on silver surfaces as NO_3_. However, theoretical calculations are in conflict with the N 1s X-ray photoelectron spectroscopy (XPS) assignment of NO_3_ adsorbed on Ag(111). Here, density functional theory calculations, ab initio thermodynamics, and core-level shift calculations, in combination with XPS measurements, are used to investigate the adsorption of H_2_ and NO_2_ on oxygen-covered Ag(111). We found that the presence of hydrogen on the surface as hydroxyl groups is thermodynamically favored and explains the experimentally observed O 1s binding energy (BE) signature on Ag(111) at 530.4 eV. Moreover, we determined that NO_2_ adsorbed in a dimer form (N_2_O_4_) elucidates the N 1s BE signature observed at 405.8 eV, whereas the N 1s BE signature for NO_3_ is predicted to be closer to 407 eV. Overall, our results suggest that the species assignment for N 1s XPS peak at 405.8 eV on O-covered Ag(111) upon exposure to NO_2_ should be reevaluated. This work demonstrates a combined ab initio/experimental methodology capable of resolving XPS spectra of a system with chemoresponsive and catalytic applications.

## Graphical abstract

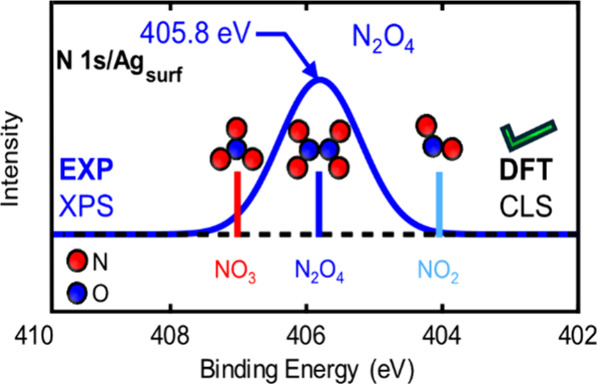

## Introduction

Strict standards have been established in most countries around the world to reduce environmental and occupation exposures to NO_2_ and protect human health.^1,2^ Reduction in emissions of harmful gases to the atmosphere is ultimately the most effective strategy to alleviate the health risks they provoke. However, timely detection of high concentrations of gases like NO_2_ can also be a means to limit transient exposure to these species and to reduce critical health problems associated with such exposure. Sensing of NO_2_ usually requires a surface where NO_2_ can be readily adsorbed and/or reacted. Recently, investigation of the adsorption or reaction of NO_2_ on metals and metal oxides has seen increased activity.^3–9^ Metal-based materials are particularly attractive as NO_2_-reactive systems due to the variety of reactions that involve NO_2_ on their surfaces.^9–19^ Among these metallic systems, silver-based materials are particularly promising.^14–19^ The interaction of NO_2_ with pristine Ag(111) has been investigated extensively.^15–18^ However, investigations on the interaction of NO_2_ with oxidized Ag(111) are rare,^19^ while studies at ambient conditions have not, to the best of our knowledge, been previously reported.

Polzonetti et al.^15,16^ studied the adsorption of NO_2_ on Ag(111) at different temperatures [*T* = 25, 90, and 300 K] and followed the evolution of the adsorbates upon annealing up to 300 K with X-ray photoelectron spectroscopy (XPS) and ultraviolet photo-electron spectroscopy (UPS). In their first work,^15^ Polzonetti et al. found that NO_2_ adsorbs dissociatively at temperatures of 90 and 300 K, forming NO and O on the surface (state I).^15^ However, it was found that increasing exposure to NO_2_ in the lower temperature regime leads to different surface reaction mechanisms upon annealing. At lower NO_2_ exposure, the adsorbate was reported to condense to a species they assigned to N_2_O_4_ [N 1s (O 1s) = 404.8 (532.7) eV] (state II), which, upon annealing, was reported to react and desorb from the surface, leaving only NO behind [N 1s (O 1s) = 401.6 (531.0) eV].

In contrast, at a higher NO_2_ exposure and 90 K, the adsorbate was reported to dissociatively adsorb as NO on the surface, with a shifted XPS signature [N 1s (O 1s) = 402.0 (531.1) eV] (state III)*,* eventually creating multilayers of N_2_O_4_ [N 1s (O 1s) = 406 (534) eV] (state II (multilayer)). Annealing to *T* *>* 140 K under high exposure conditions led to the formation of a third species, with a core–electron binding energy (BE) of N 1s (O 1s) = 405.8 (531.8) eV, which was assigned to NO_3_ on the surface (state IV). The assignment of the reported NO_3_ species in ref 15 was based on the stoichiometry of the observed O 1s and N 1s peaks (*∼*3:1).

This assignment was additionally referenced to a claimed assignment of nitrate at a similar energy on iron (Fe), attributed to Brundle.^20^ In fact, Brundle cites the work of Honda and Hirokawa,^21^ where the adsorption of NO is studied on an Fe system and the assignment of nitrates is actually given to a N 1s binding energy of 407.1 eV. In ref 20, Brundle reports NO_2_ exposure on nickel films.^20^ Moreover, we found that Brundle specifically reports not finding any NO_3_^*–*^ when investigating the adsorption of NO_2_ on nickel.^20^

In their follow-up work,^16^ Polzonetti et al. followed a similar methodology as they did in their original work and studied the adsorption of NO_2_ on pristine Ag(111) at 25 K (to 300 K). Their findings are somewhat similar to their earlier work, but they also found a fifth state (state V) that could be observed at intermediate temperatures if a thick-enough layer of N_2_O_4_ was formed at the lowest temperature regime (*T* = 25 K). This reported species was assigned to condensed monomeric NO_2_, with a core–electron BE of N 1s (O 1s) = 405.8 (531.4) eV.^16^

Adsorption of NO_2_ on O-covered Ag(111) has been reported more recently in ref 19, where the adsorption was characterized with the use of reflection absorption infrared spectroscopy (RAIRS) at various temperatures (*T* *>* 300 K) and various NO_2_ exposures (6, 90, 180 L), as well as with temperature-programmed reaction spectroscopy (TPRS). On the basis of vibrational frequencies, the analyte was noted to be adsorbed on the oxidized surface as an NO_3_ species.^19^

The different adsorbed species on the surface of the pristine Ag(111) (NO) and O-pre-exposed Ag(111) surface (NO_3_) at 300 K reported respectively in refs 15 and 19 suggest that oxygen coverage plays an important role on the adsorption mode of NO_2_ on silver. Additionally, one must note a troubling issue regarding the XPS assignment in ref 16 for NO_2_ (state V) to the N 1s BE of 405.8 eV, as NO_3_ (state IV) is also assigned to the same N 1s BE. Interestingly, physisorbed NO_2_ has been reported in investigations of NO_2_ adsorption on Cu_2_O, which was assigned to a N 1s BE of *∼*406 eV,^6^ suggesting that the assignment in refs 15,16 to adsorbed NO_3_ (state IV) might need to be revisited. To add to the confusion on this N 1s assignment on silver to the species with a BE of 405.8 eV, the reaction of NO and O on Ag surfaces is known to lead to the formation of an overlayer of AgNO_3_ (*∼*20 Å), with a N 1s (O 1s) BE of 406.2 (532.2) eV.^22–25^

Few theoretical calculations on the N 1s core–electron binding energy exist for NO_*x*_ (NO, NO_2_, and NO_3_) species adsorbed on silver surfaces.^26^ The calculations were performed by creation of a core–electron hole on the N 1s state, assuming full screening of the core hole, under the Perdew, Burke, Ernzerhof formulation of the exchange-correlation energy. In ref 26, using a combined experimental/theoretical approach, Klacar et al. studied the adsorption of NO on a p(4×4)-Ag(111) surface and calculated the N 1s BE of NO_2_, NO_3_, and N_2_O_4_ to be 403.8, 406.5–406.7, and 405.4 eV, respectively. Interestingly, Klacar et al. assigned NO_3_ N 1s to the measured BE of 405.8 eV, following the assignment in the literature.^15^ However, the calculated N 1s BEs reported in ref 26 are in fact in agreement with other experimental assignments for NO_2_ N 1s at 404 eV^6^ and NO_3_ at 407 eV,^9^ further suggesting that the assignment of NO_3_ to the N 1s BE of 405.8 eV on O-precovered Ag(111) could have been mistaken and highlighting the need for a systematic study of the adsorption of NO_2_ on pristine and on O-precovered Ag(111).

The challenges associated with assignment of experimental XPS spectra to surface-adsorbed species are numerous, particularly when clear experimental reference systems are lacking or when surface reactivity leads to multiple possible reaction products. As a result, pre-existing XPS assignments found in the literature often serve as the primary source of insight into the adsorbates’ nature. While computational investigations from first principles can provide valuable clarification, they require careful consideration of surface models, adsorbate variations, and coverage effects to ensure accurate assignments. Successful implementations of computational methodologies for XPS simulation have recently been demonstrated in the reinterpretation of the O 1s BE assignment (530–532 eV), often associated in the literature with oxygen vacancies on various oxides, as arising instead from surface hydroxyl groups.^27–29^ The integration of computational strategies with experimental observations not only improves the reliability of XPS assignments but also facilitates improved understanding of catalytic reaction mechanisms and potentially improved catalyst design. More specifically in the context of the present work, combining computational strategies with experimental measurements can shed new light on the identity of reactive intermediates on silver surfaces and aid in the design of chemoresponsive Ag-based systems for the detection and reaction of NO_2_ under ambient conditions.

Here, we use density functional theory (DFT) calculations and ab initio thermodynamics, together with X-ray photoelectron spectroscopic (XPS) measurements, to investigate the adsorption of H_2_ and NO_2_ on an oxygen-precovered silver thin-film surface. The DFT calculations are performed on pristine and oxidized Ag(111), whereas the experimental measurements are carried out on an oxygen-precovered silver surface supported on a gold substrate, fabricated by electrochemical deposition. Our results suggest that hydrogen adsorption and formation of hydroxyl groups on O-precovered Ag(111) is thermodynamically favorable. O 1s core-level shift (CLS) calculations on these adsorbed hydroxyl groups elucidate the experimentally observed spectra. Additionally, our results suggest that NO_2_ adsorbs on the surface in a dimer state (N_2_O_4_), with a calculated N 1s and O 1s BE of 405.8 and 532.0 eV, respectively, in excellent agreement with our experimental observations. We find that the N 1s CLS of N_2_O_4_ is unaffected by the presence of surface hydroxyl groups, whereas the O 1s CLS can be shifted by up to 0.4 eV to lower BE (531.6 eV) with increasing OH coverage.

## Methods

### Sample Preparation

#### Preparation of Gold Substrates

Semitransparent films of gold with thicknesses of 200 Å were deposited onto piranha-cleaned glass slides (see details in the Supporting Information (SI)) using an electron-beam evaporator (VEC-3000-C manufactured by Tek-Vac Industries, Brentwood, New York).^30^ A layer of titanium with a thickness of 20 Å was employed to enhance adhesion between the glass microscope slides and the gold films. The deposition rates for both gold and titanium were 0.2 Å/s. The pressure in the evaporator was kept below 3 × 10^*–*6^ Torr throughout the deposition process. The predominant crystallographic face of the polycrystalline Au substrate deposited under vacuum is Au(111).^31^ Optically reflective films of gold used for infrared (IR) and X-ray photoelectron spectroscopy (XPS) were prepared by sequential deposition of 100 Å of titanium and 1000 Å of gold onto silicon wafers.

#### Preparation of Ag Surfaces

Ag overlayers on Au were prepared via electrodeposition onto gold films using an aqueous solution containing 0.1 M HClO_4_ + 1 mM AgClO_4_.^32^ All electrochemical experiments were performed using a Pine Instruments AFCBP1 bipotentiostat (Grove City, Pennsylvania). The electrochemical cell was assembled in a standard three-electrode configuration using a gold film as the working electrode, a platinum wire mesh as the counter electrode, and a silver chloride electrode as the reference electrode (BASi, West Lafayette, Indiana). For submonolayer deposition of Ag, a voltage of 410 mV was used. Consistent with a process of underpotential deposition at 410 mV, following an initial transient current, we observed the current density to decay to zero. Prior studies have reported the growth of Ag adlayers on Au during underpotential deposition to occur via 2D rather than 3D growth (see the Supporting Information).^33–35^ In experiments performed to prepare thicker Ag overlayers on Au, the charge density passed was used to determine the Ag coverage: One monolayer (ML) equivalent was defined as 220 μC/cm^2^ for Ag on Au(111).^36^ After deposition, each Ag/Au electrode was removed from the electrochemical cell and rinsed for 2 min with flowing Milli-Q water.

#### XPS Measurements

X-ray photoelectron spectroscopy (XPS) was performed using a Scienta Omicron ESCA-2SR instrument with an operating pressure of approximately 10^*–*9^ Torr. Monochromatic Al Kα X-rays (1486.6 eV) were used, and photoelectrons were collected from a 5 mm-diameter analysis area. The photoelectrons were collected at a 0° emission angle, with a source-to-analyzer angle of 54.7°. Survey scans were performed with a pass energy of 200 eV, while high-resolution scans were conducted using a 50 eV pass energy. The samples were found to be conductive, and no charge neutralization was necessary. All scans were collected at 200 ms dwell times. All XPS results presented in this manuscript were analyzed by CasaXPS software. A Gaussian–Lorentzian function was assumed for the oxygen and nitrogen components with a linear background, while an asymmetrical Voigt function was utilized for metal components with a Shirley background. This approach has been successfully employed in previous studies.^30,37,38^ The evolution of the oxygen and NO_2_ adsorption was monitored before and after dosing 10 ppm of NO_2_ (balanced by N_2_ at 1 atm) at 298 K to the reaction chamber. The NO_2_-exposure XPS measurements were reproduced on a 25 Ag-ML thick system, with the same findings as for the thinner system (Figure S1).

#### DFT Calculations

Density functional theory (DFT) calculations were performed using the Vienna Ab initio Simulation Package (VASP).^39–42^ The exchange-correlation energy is calculated using the Perdew, Burke, Ernzerhof (PBE) formulation,^43^ while dispersion interactions are approximated within the Grimme-D3 method at zero damping.^44^ The Kohn–Sham orbitals were expanded with the use of a plane wave basis, truncated at a kinetic energy of 400 eV. The interaction between core and valence electrons was described with the use of projector augmented wave potentials (PAW).^45,46^ Hydrogen, nitrogen, oxygen, and silver were described with 1, 5, 6, and 11 valence electrons, respectively. The integration of the Brillouin zone was performed with finite sampling centered around the Gamma point. A *k*-point mesh of 4×4×1 [2×2×1] was used for the surface calculations. The energy was considered to be converged when the energy difference between electronic steps was smaller than 10^*–*6^ eV. Structural optimization was performed with the limited-memory Broyden–Fletcher–Goldfarb–Shanno (LBGS) method as implemented with the Henkelman tools for VASP.^47^ Structural optimization was considered converged when the forces acting on the atoms were smaller than 0.02 eV/Å. Activation energy of H_2_ dissociation was calculated using the climbing-image nudge elastic band (CI-NEB) method.^48^ To describe the minimum energy path, seven images were used along the reaction coordinate. The saddle points were confirmed by vibrational analysis, where only one imaginary frequency was found. The vibrational analysis was performed using finite differences with displacements of 0.015 Å.

Oxidation of Ag(111) was investigated at different levels of oxygen coverage on two different surface cells (see Figure 1). Initial oxidation was investigated on a four-layer-thick Ag(111) slab with a (4×4) supercell. Reconstruction to the p(4×4)-Ag(111) surface structure was described with a total of five layers, also with a (4×4) surface cell.^26^ A greater degree of oxidation was interrogated using the p(7×7) reconstruction. This further oxidized surface reconstruction was composed of 2 layers of Ag_2_O­(111) in a p(3×3) surface cell repetition, supported on a four-layer-thick Ag(111)-(7×7) supercell (Figure S3).^49,50^ The atoms in the bottom two Ag layers for all slab models were constrained to their bulk positions during structural optimization. All periodic images of slabs models were separated by at least 20 Å of vacuum in the *z*-direction. Energy calculations for the gas-phase species were performed in a 11×12×13 Å^3^ simulation box. The H–H and N–O bond lengths of H_2_ and NO_2_ in gas-phase were calculated to be 0.75 and 1.21 Å, respectively. Both are in good agreement with the respective experimental values (0.74 and 1.19 Å). Spin unrestricted calculations for ionic optimizations were performed in all cases.

**Figure 1:**
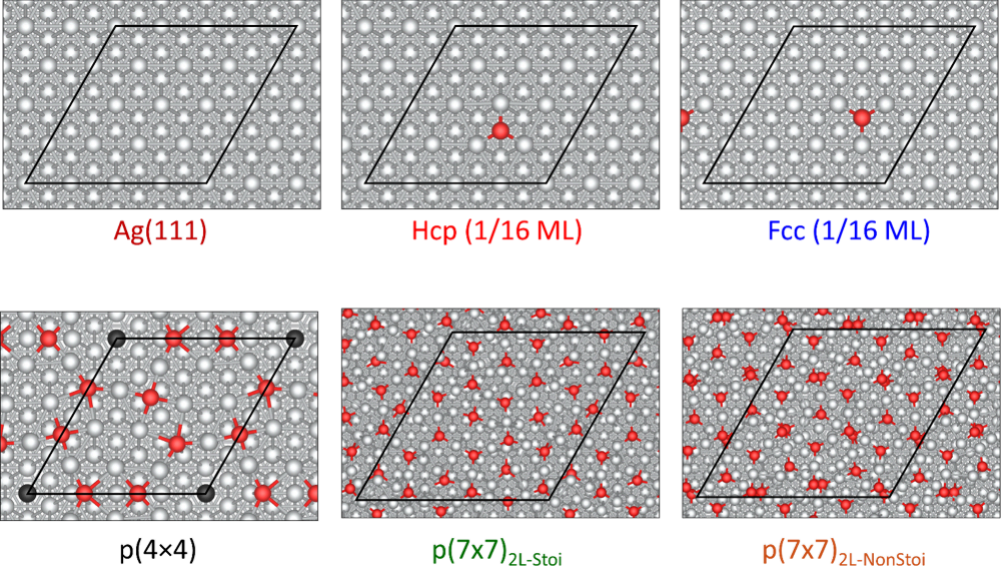
Top views of structural models of oxidized Ag(111) surfaces under different oxygen-surface coverages. The Ag-vacancy site is highlighted by showing the subsurface Ag atom in black. Atomic color code: Ag (silver), O (red), sub-Ag-vacancy site (black/dark gray). The surface unit cell is indicated with black lines. A ball-and-stick model is used to show the atomic structures.

To elucidate an appropriate oxidized surface model for NO_2_ adsorption, the relative stability of the differently oxidized Ag(111) was evaluated under relevant ambient conditions (*T* = 300 K). To do this, we calculated the change in surface free energy (Δγ­(*T, p*)) as a function of oxygen chemical potential (μ_O_(*T*, *p*)) using the formalism of ab initio thermodynamics.^51,52^ Similarly, the stability of H_2_ adsorption on the p(4×4)-Ag(111) surface reconstruction was also evaluated as a function of the H_2_ chemical potential (μ_H2_ (*T, p*)). The change in surface energy as a function of adsorbate chemical potential is expressed as

$$\Delta \gamma \left(T,p\right)=\frac{1}{A}\left[E_{ads/surf}-E_{surf}-N_{Ag}\mu_{Ag}-N_{ads}\mu_{ads}\left(T,p\right)\right]$$ 1

where *A* is the area of the corresponding surface cell. *N*_ads_ is the number of adsorbates in the surface unit cell (O or H_2_). *N*_Ag_ is the number of silver atoms transferred from a bulk thermodynamic reservoir onto the surface and is only relevant for the reconstructed surfaces in the case of oxygen adsorption. The third term, *N*_Ag_μ_Ag_, accounts for the differences in the number of silver atoms between the slabs, when we compare oxygen adsorption between pristine Ag(111), p(4×4)-Ag(111), and the p(7×7)-reconstruction, with *N*_Ag_ = [0, 12, 72], respectively. *E*_ads/surf_ is the energy of the entire slab/adsorbate system, while *E*_surf_ is the energy of the slab without adsorbates. For the case of oxygen adsorption, *E*_surf_ is the energy of the pristine Ag(111). For the adsorption of H_2_, *E*_surf_ is the energy of the p(4×4)-Ag(111) surface reconstruction. μ_Ag_ represents the chemical potential of silver, calculated as the energy of an Ag atom in the bulk. Finally, μ_ads_(*T*, *p*) is the chemical potential for the adsorbate (O or H_2_) at the respective temperature and pressure (*T*, *p*). The corresponding adsorbate chemical potential of oxygen is calculated as

$$\mu_{O}\left(T,p\right)=\frac{1}{2}\left[E_{O_{2}}+E_{O_{2}}^{ZPE}+{\mu^{’}}_{O_{2}}+k_{B}T\ln\left(\frac{p_{O_{2}}}{p^{0}}\right)\right]$$ 2

where the factor of 1/2 is used since the chemical potential is expressed in terms of the O_2_ molecule. *E*_O_2__ is the calculated total energy of the O_2_ molecule at 0 K, while *E*_O_2__^ZPE^ is its calculated zero-point energy. μ′_O_2__ is the reference chemical potential of O_2_ calculated from thermodynamic tables with respect to the target temperature (*T*) at standard pressure.^53^ The logarithmic term in eq 2 accounts for the effect of pressure on entropy contributions of the gas-phase species, with respect to the standard state. The chemical potential of molecular hydrogen (μ_H_2__(*T*, *p*)) is calculated accordingly. To study the adsorption of N_*x*_O_*y*_ on an O-precovered Ag(111), we calculated the average adsorption energy as

$$E_{b}=\frac{1}{N_{adsor}}\left[E_{adsor/surf}-E_{surf}-N_{adsor}E_{adsor}\right]$$ 3

Here, a negative value indicates exothermic adsorption. In this case, *N*_adsor_ represents the number of absorbed species per surface unit cell. *E*_adsor/surf_ is the total energy of the adsorbate/slab system, *E*_surf_ is the energy of the respective slab, and *E*_adsor_ is the gas-phase energy of the adsorbed species.

Theoretical X-ray photoelectron binding energies, core-level shifts (CLS), are calculated with the use of a double-reference methodology. Atomic N and atomic O in the bulk-constrained layers of the surface systems are used as reference for the N 1s and O 1s CLS. Using the experimentally known values of the N 1s BE for NO adsorbed on p(4 × 4)-Ag(111),^26^ and O 1s BE for the oxygen atoms on the p(4 × 4)-Ag(111) itself, we calculated the corresponding shift in energy of the atomic reference species, allowing us to reference our CLS calculations with absolute values of experimentally obtained core–electron binding energies. The CLS are calculated with respect to the atomic species in the bulk as

$$E_{CLS}=\left[E^{*}-E^{0}\right]-\left[E_{ref}^{*}-E_{ref}^{0}\right]$$ 4

Here, E* is the energy of the core-ionized system of interest. *E* ^0^ is the unperturbed ground-state energy of the same system. *E*_ref_^*^ is the energy of the core-ionized atomic species (N or O) in the bulk-constrained layer of the surface. *E*_ref_^0^ is the ground-state energy of the unperturbed system. Since we have placed the reference atomic species in the same surface structure for all corresponding calculations, the ground-state energies effectively cancel out. The core-level shifts are calculated under the final-state approximation and are evaluated with the use of a PAW potential where complete screening of the core hole is assumed.^54^ Charge neutrality is ensured by adding an extra electron to the valence. This procedure is known to be appropriate for systems without a band gap.^55,56^ The convergence of the N 1s CLS with respect to the number of silver layers was corroborated on an eight-layer system, well within 0.1 eV for the tested adsorbates. The N 1s BE energy difference, the result of different charge-compensation methods (jellium background or addition of an electron to the valence), was found to be less than 0.1 eV for the adsorbate we evaluated (N_2_O_4_), well within the expected accuracy for this type of calculation. A discussion on the CLS calculations is presented in the Supporting Information, section ‘Core-level shifts: Assumptions and Limitations’. Charge analysis is performed on the structures of interest with the use of Bader charges, as implemented with the code developed by Henkelman and co-workers.^57^ All structures shown in the manuscript were prepared using VESTA software.^58^

## Results

### X-ray Photoelectron Spectroscopy

The adsorption of NO_2_ on a 1.13 ML Ag/Au surface was characterized by XPS measurements of the N 1s and O 1s core–electron binding energy (BE) before and after exposure to 10 ppm of NO_2_ (balanced by N_2_ at 1 atm) at a temperature of 298 K (Figure 2). For the N 1s spectra (Figure 2a), no discernible signature is observed before exposing to NO_2_. Upon exposure to NO_2_, a single peak is observed at a BE of 405.8 eV. The N 1s BE peak at 405.8 eV is consistent with prior reports, where it was assigned to adsorbed NO_3_.^15,16^ From the O 1s spectra (Figure 2b), before NO_2_ exposure, the XPS spectrum was deconvolved into two peaks at 530.4 and 531.9 eV. The lower energy peak (530.4 eV) corresponds to surface oxygen and has been assigned to surface hydroxyls groups,^50,59,60^ while the peak at higher energy (531.9 eV) has been assigned to adventitious carbon compounds.^61^ The ratio between the surface oxygen O 1s peak (530.4 eV) and Ag 3d5/2 intensity was measured to be approximately 0.35, which is close to the O coverage of the p(4 × 4)-reconstructed structure on the Ag(111) surface at room temperature.^62^ Upon exposure to NO_2_, a peak appears at a O 1s BE of 532.0 eV and is thus associated with the adsorbed NO_*x*_ species. Additionally, the surface oxygen peak at 530.4 eV reduces slightly in intensity, suggesting that some surface oxygens are being consumed by reaction with the adsorbate. Importantly, all peaks persisted in the XPS measurements under high vacuum, indicating the irreversible adsorption of NO_2_ to the Ag surface. The stability and core–electron binding energy of NO_*x*_ adsorbates were investigated with DFT calculations.

**Figure 2:**
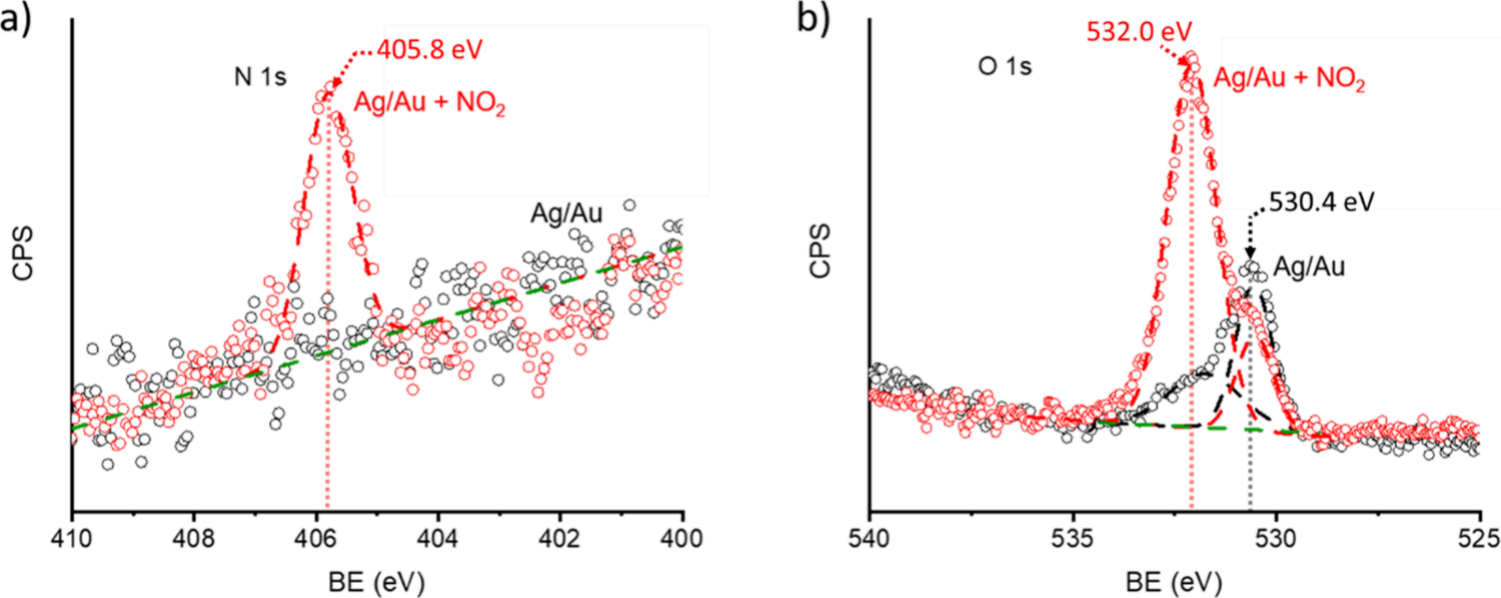
Photoelectron emission spectra of a 1.13 ML Ag/Au surface before (black) and after (red) exposure to 10 ppm of NO_2_ (balanced by N_2_ at 1 atm) at a temperature of 298 K. (a) N 1s spectra. (b) O 1s spectra. Decomposed peaks are shown as dashed lines. Baseline correction is shown in green.

### Surface Model and Surface Stability

To determine a relevant surface model for NO_*x*_ adsorption, the degree of surface oxidation was investigated first with DFT calculations. The adsorption of oxygen on Ag(111) was studied under three oxygen coverage conditions, investigated with five different overlayer structures. Namely, adsorption was studied at (i) *low oxygen coverage* (1/16 ML) on the Ag(111) surface, exemplified by adsorption of a single oxygen at an FCC site, as well as at an HCP site. (ii) *intermediate oxygen coverage*, where the p(4×4)-surface reconstruction was used as a model system. (iii) *high oxygen coverage*, where a thin overlayer of Ag_2_O begins to form on the surface, investigated with the use of a p(7×7) reconstruction at stoichiometric and nonstoichiometric conditions.^49,50^ All surface structures are shown in Figure 1. The p(4×4) surface reconstruction is described with four layers of pristine Ag(111) and an overlayer of 12 silver ions and six surface oxygen atoms. The Ag ion overlayer is arranged in the shape of two triangles of the same size, each respectively occupying hcp and fcc sites over the Ag(111) substrate, separated by two oxygen atoms on each side of the triangles, each characterized by a 4-fold occupation. The formation of the two triangles creates a Ag vacancy on the topmost layer, at the center of a hexagonal Ag rim, shown in Figure 1 by coloring the Ag ion right below the vacancy in black. The p(7×7) reconstruction is described as the adsorption of two layers of p(3×3)-Ag_2_O­(111), supported on a four-layered p(7×7)-Ag(111) substrate, as reported in ref 51. To guarantee that the optimal overlayer configuration stacking was being probed, the p(7×7) reconstruction was optimized from the reported structure by displacing the Ag_2_O overlayer over the *xy*-plane with respect to the Ag(111) substrate. Further description of the procedure and optimized structure can be found in Figure S3.

We have chosen the Ag(111) as a surface model for our ab initio calculations because Ag(111) is the most thermodynamically stable surface orientation and therefore most likely to dominate the surface of our sample. Determining the correct surface structure of oxygen-modified Ag(111) with increasing oxygen coverage is an open topic of investigation.^49,50,63^ Here, we have chosen the widely reported p(4×4) and p(7×7) surface structures as model systems that represent slightly different degrees of surface oxidation.^64–66^ It must be noted that other single-crystal surface structures, like the lightly more oxidized c(4×8) or the p­(4×5 $\sqrt{3}$ )­rect, also exist in the literature as phases that can coexist during silver oxidation.^50,63,66^ However, the p(4×4) and p(7×7) surface structures have a surface Ag/O ratio similar to those experimentally determined by our XPS measurements and have been suggested before to be realistic models for moderate (θ_O_ < 0.4 ML) Ag(111) oxidation.^49,50,64–67^

The stability of the different surface structures upon oxygen adsorption was determined by calculating their surface free energy as a function of oxygen chemical potential with the use of ab initio thermodynamics (Figure 3).^51,52^ The chemical potential of oxygen (Δμ_O_) was converted to its corresponding partial pressure at *T* = 298 K and *T* = 498 K and plotted at the top axis of Figure 3. From this figure, we see that the surface oxidizes from the pristine, to the p(4×4) reconstruction at an oxygen chemical potential (Δμ_*O*_) of −0.66 eV, corresponding to a partial pressure of *p* = 10^*–*7^ atm at 298 K, the relevant reaction temperature for our experimental measurements. We predict further oxidation to the stoichiometric p(7×7) reconstruction at a Δμ_O_ = −0.14 eV (*p* ∼ O­(10^2^) atm), in agreement with previous reports.^50^ Our results suggest that the experimental Ag/Au surface is expected to be moderately oxidized. Moreover, p(4×4) exhibits a similar oxygen coverage to one inferred from the experimental XPS measurements (I­[O 1s]/I­[Ag 3d 5/2] ∼ 0.35). Thus, the p(4×4) reconstruction is likely an appropriate model system to capture silver surface oxidation under our experimental conditions.

**Figure 3:**
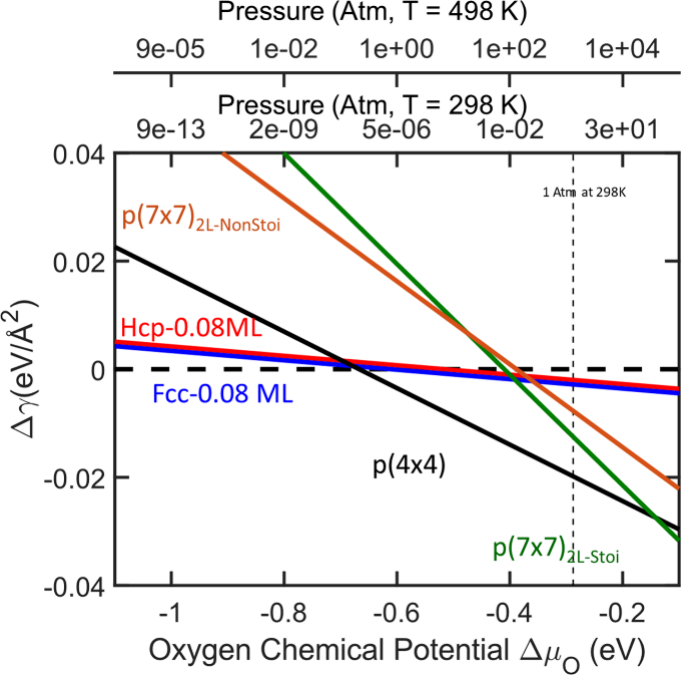
Surface stability of Ag(111) as a function of oxygen chemical potential (Δμ_O_). The corresponding pressure is plotted on the top axis at *T* = 298 K and *T* = 498 K. The pristine Ag(111) surface is shown as a dashed line at zero Δγ energy. Labels are color-coded in accordance with Figure 2.

### Hydrogen Adsorption

The origin of the O 1s features at binding energies between 530.0 and 530.7 eV on oxidized Ag(111) has been debated.^68^ Different species have been assigned to measurements of the O 1s BE on this energy range, including subsurface oxygen,^69^ disordered surface oxygen,^70^ and hydroxyl groups.^50,59,68^ The O 1s BE of surface hydroxyl has been calculated before to be 530.0 eV on the p(4×4) reconstruction,^50^ in relatively good agreement with experimental observations. This finding suggests that hydroxyl groups could be indeed responsible for the observed O 1s features, where the energy spread observed in the different experimental observations could be caused by different coordination of the hydroxyl groups, or due to coverage effects. To elucidate the nature of the observed O 1s peak at a BE of 530.4 eV (Figure 2), we investigated the effect of the degree of surface hydroxylation on the p(4×4) reconstruction. Elucidating the stability of hydrogen adsorption on the p(4×4) reconstruction was done following the same approach as for surface oxidation, but with respect to the hydrogen chemical potential (μ_H_2__(*T*, *p*)).

### Surface Stability

The stability of hydrogen adsorption on the p(4×4) reconstruction was considered under six hydrogen coverages, from 1 to 6 H, increasing coverage by adding one individual H atom in the unit cell each time (Figure 4). We restricted the investigation of hydrogen adsorption on the surface O ions, in order to investigate only surface hydroxyls. The adsorption of H on Ag was explored at the low coverage limit on 3 nonequivalent Ag sites, all converging to H adsorption at the center of its corresponding Ag_6_ structure, in the hcp site. We calculated the adsorption energy of H on Ag to be endothermic by 0.28 eV with respect to 1/2 *H*_2_(*g*), suggesting that H adsorption takes place practically exclusively on O sites. From Figure 4, we observe that there is no particular ordering regarding the subsequent adsorption of hydrogen, suggesting that release of surface strain might reduce the possibility of hydrogen bonding between the surface hydroxyls. Additionally, we find that the surface hydroxyls pop up from their initial position, entrapped in the Ag overlayer. Looking at the 6 H surface structure, where all oxygen sites are decorated with H, three oxygen ions are displaced in the *z*-direction away from the surface by 0.31 Å while the rest move outward by 1.16 Å with respect to their position in the clean p(4×4)-surface reconstruction. Importantly, the oxygen ions that moved outward by 1.16 Å upon full hydrogen adsorption were located in a slightly subsurface position before hydroxylation. In comparison, for the adsorption of 1 H, the O in the formed hydroxyl is displaced by 0.47 Å with respect to O’s original position. We calculated the average hydrogen adsorption energies for 1 to 6 H on the specific surface to be −2.15, −2.49, −2.56, −2.57, 0.56, and −0.01 eV, respectively, with respect to H_2_ in the gas phase.

**Figure 4:**
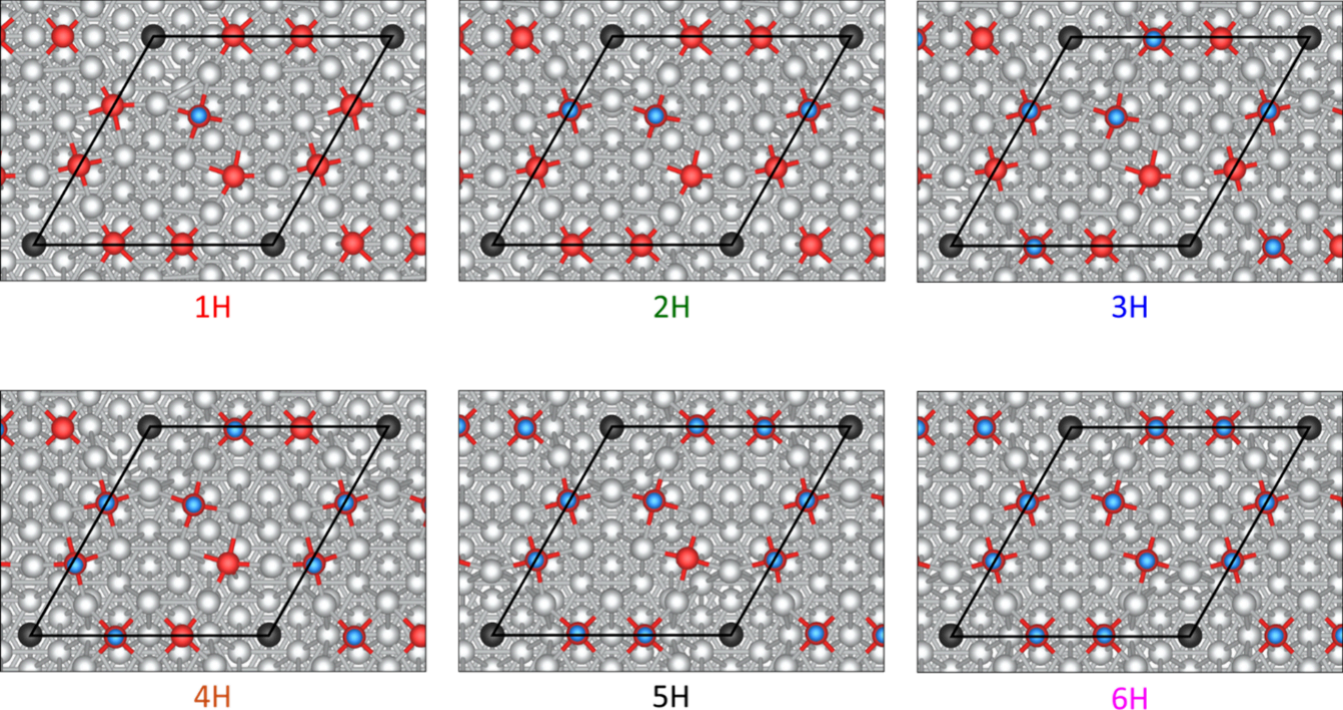
Top views of structural models for hydrogen adsorption on a p(4×4)-Ag(111) at different hydrogen surface coverages. The Ag-vacancy site is highlighted by showing the subsurface Ag atom black. Atomic color code: Ag (silver), O (red), sub-Ag-vacancy site (black), H (light blue). Surface unit cell is indicated with black lines. A ball-and-stick model is used to show the atomic structures.

The stability of the hydrogen-covered p(4×4) reconstruction as a function of hydrogen chemical potential at *T* = 298 K is shown in Figure 5. The surface free energy change is calculated with respect to the undecorated p(4×4) reconstruction, indicated in the figure as the dashed line at zero energy. The ambient hydrogen pressure (10^*–*7^ atm) is marked in the figure with a vertical black line. From the surface phase diagram in Figure 5, we note that the surface is predicted to be hydroxylated to a 4 H coverage, for all the tested hydrogen chemical potential values. This finding suggests that the oxygen species evident in experimental XPS measurements likely include hydroxylated species. We further investigated the dissociation of H_2_ on the p(4×4) reconstruction on the clean surface, and on the 2 H-covered surface. The potential energy diagram and transition state are shown in the Supporting Information (Figure S6). We find that the activation of H_2_ takes place heterolytically, over an O and an Ag site. The associated activation barriers are calculated to be 0.63 eV on the pristine p(4×4) reconstruction and 0.56 eV on the 2 H-covered surface. These barriers are significantly lower than the reported value on the pristine Ag(111), which is approximately 1.15 eV at a similar level of theory.^71,72^ Moreover, the dissociative adsorption of H_2_ and formation of a OH–OH configuration on the surface is overall exothermic by −2.49 eV on the clean and −2.66 eV on the 2 H-covered surface, suggesting that the reaction is strongly driven by thermodynamics.

**Figure 5:**
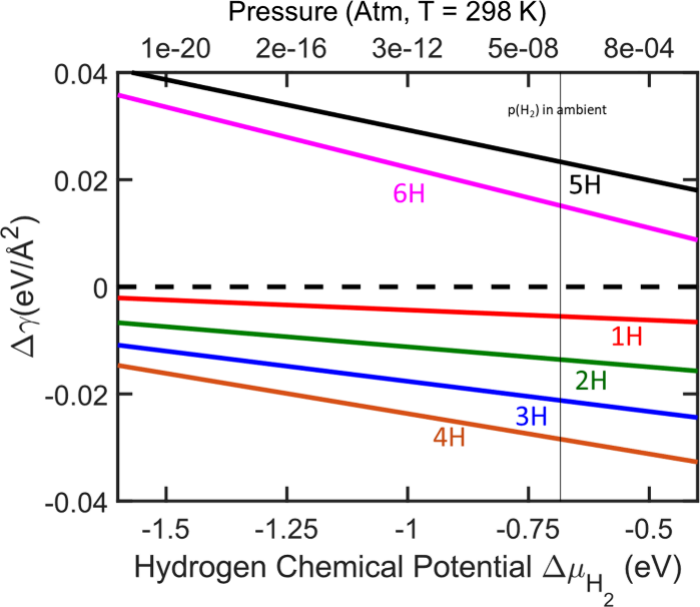
Surface stability of p(4×4)-Ag(111) as a function of hydrogen chemical potential (μ_H_2__(*T*, *p*)) at a temperature of 298 K. The pristine p(4×4)-Ag(111) is shown as a dashed line at zero Δγ energy. The pressure of ambient concentration of hydrogen in the atmosphere (10^*–*7^ atm) is shown as a vertical black line. Labels are color-coded in accordance with Figure 4.

### Surface O 1s Core-Level Shift Calculations

To elucidate the origin of the experimentally observed surface states, we calculated the O 1s core-level shift (CLS) for nine oxygen-surface structures of interest. Namely, we investigated the CLS for the HCP (1/16 ML), FCC (1/16 ML), subsurface O (1/16 ML), p(4×4) reconstruction, *y*H/p­(4×4) reconstruction (*y* = 2, 4, 6), and the stoichiometric and nonstoichiometric p(7×7) reconstructions. Results are shown in Figure 6.

**Figure 6:**
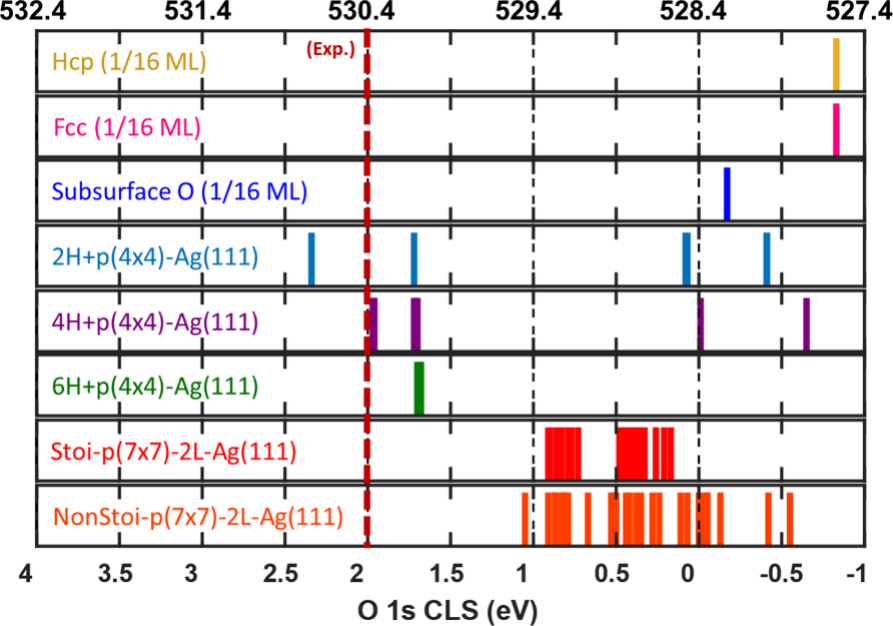
Calculated O 1s core-level shifts for oxygen and hydroxyl covered Ag(111). The bottom axis shows the relative shift with respect to the reference. The top axis shows the calculated absolute energies referenced to the known values of O in pristine p(4×4)-Ag(111). The experimentally observed energy is shown as a red dashed line. The labels in the figure correspond to the labels used for the O-covered structures in Figure 1 and the H/p(4×4) structures shown in Figure 4. Vertical lines in black are there to guide the eye, connecting the bottom axis (CLS) with the top axis (O 1s BE).

The CLS were calculated with respect to an O ion located in an octahedral site in the bulk-constrained layers in its respective simulation slab. The calculated O 1s CLS for the reference O ion with respect to the oxygen overlayer on the p(4×4)-Ag(111) resulted in a CLS of 1.2 eV. The O 1s BE for the O atoms on the p(4×4)-Ag(111) is known to be 528.4 eV,^68^ resulting in a value of 529.6 eV for the O 1s BE of atomic O in the bulk of Ag(111). The relative shift values are presented in Figure 6 with respect to the p(4×4) reconstruction. This double-reference system allows for a simple noninteracting reference that translates relative energy shifts to total core–electron binding energies. We found a ±0.07 eV energy spread for the O ions on the p(4×4)-Ag(111), suggesting that our CLS calculations are accurate within 0.1 eV. We calculated the O 1s BE for the HCP (1/16 ML), FCC­(1/16 ML), and subsurface O to be 527.6, 527.6, and 528.2 eV, respectively. Our results for the bulk O reference, with an associated O 1s BE of 529.6 eV, suggest that, at low coverage, disordered surface oxygen or subsurface oxygen cannot rationalize the experimental feature observed at 530.4 eV. For the p(7×7) reconstruction, where a higher degree of subsurface oxidation is considered, we found that the stoichiometric and nonstoichiometric structures exhibit a spread of O 1s BEs, ranging from 528.6 to 529.3 eV for the former, and from 527.8 to 529.4 eV for the latter. Finally, we calculated the O 1s BE for the 2, 4, and 6 H on p­(4*×*4) reconstruction. Interestingly, the formed OH groups exhibit only three different values for the calculated O 1s CLS, despite the different coverages tested. For the 2H structure, the calculated O 1s BE were 530.7 and 530.1 eV. Increasing the hydrogen coverage to 4 H resulted in two Os with an O 1s BE of 530.4 eV while the other two exhibited an O 1s BE of 530.1 eV. Finally, the O ions in the 6 H configuration all showed the same O 1s BE of 530.1 eV. Notably, on both the 4 H and 6 H configurations, the undecorated O ions either maintain their p­(4 × 4) O 1s BE or shift slightly to lower binding energies. Nevertheless, the calculated O 1s BE for the hydroxylated surfaces can rationalize the experimental observations. Particularly, the thermodynamically stable hydrogen coverage, 4 H/p(4×4) reconstruction, explains the surface oxygen peak (O 1s BE = 530.4) in Figure 2.

### NO_2_ Adsorption

We then studied the adsorption of NO_2_, and of possible reaction products, on the pristine and oxidized silver surfaces. The coverage of hydrogen was first neglected on the reconstructed surface, to simplify the search for stable adsorbates. The analytes assessed were, namely, NO, NO_2_, NO_3_, N_2_O, N_2_O_3_, N_2_O_4_, and N_2_O_5_. We first determined that N_2_O, N_2_O_3_, and N_2_O_5_ desorb or decompose upon adsorption on the p(4×4) reconstruction (see Figures S4 and S5). The preferred adsorption configurations for NO, NO_2_, NO_3_, and N_2_O_4_ are presented in Figure 7. Notably, the adsorption of N_2_O_4_ can take place over the Ag vacancy, parallel to the surface and ∼2.48 Å above it. Similarly, NO, NO_2_, and NO_3_ are also adsorbed in the Ag vacancy, with NO_2_ and NO_3_ occupying the defected site with an O ion, while NO is adsorbed by nitrogen down in a slightly tilted configuration. The case of NO_2_ adsorption on an O site was also evaluated, despite being less stable (by 0.3 eV) than adsorption on the Ag vacancy. A NO_3_-reconstructed surface structure was also investigated, as reported in ref 26, where three NO_3_ are entrapped in the topmost layer of a p(4×4)-like reconstruction, to simulate the formation of a layer of AgNO_3_.^25,26^

**Figure 7:**
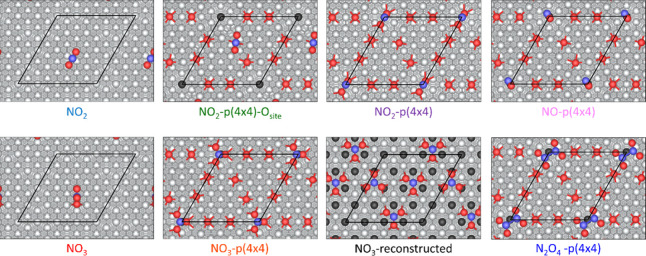
Top views of structural models of N_*x*_O_*y*_ adsorption on pristine Ag(111) and p(4×4)-Ag(111). The Ag-vacancy site is highlighted by showing the subsurface Ag atom in black. Atomic color code: Ag (silver), Sub-Ag-vacancy site (black), O (red), and N (blue). The surface unit cell is indicated with black lines. For the NO_3_-reconstructed structure, the first sublayer is shown with Ag ions colored in black. A ball-and-stick model is used to show the atomic structures.

To determine the likelihood of finding each adsorbate on the surface, we calculated the adsorption energy of each analyte with respect to its gas-phase reference. On the pristine Ag(111), we found the adsorption energies of NO_2_ and NO_3_ to be −1.44 and −2.18 eV, respectively, while we did not find a stable configuration for N_2_O_4_ on that surface. The adsorption energies for NO, NO_2_ (NO_2_–O_site_), NO_3_, and N_2_O_4_ on the p(4×4) reconstruction were calculated to be −0.62, −1.21, (−0.97), −2.41, and −0.54 eV, respectively. Finally, the adsorption of NO_3_ in the reconstructed surface was calculated to be exothermic by −3.61 eV, with respect to N*O*_2_(*g*)+ 1/2 *O*_2_(*g*).

### Adsorbate N 1s and O 1s Core-Level Shift Calculations

We calculated the N 1s core-level shift for the adsorbed species following the same double-reference approach. The calculations were performed with respect to N ion placed in an octahedral site in the bulk-constrained layers of the Ag(111) surface. The calculated N 1s CLS for the N reference with respect to NO adsorbed on the p(4×4)-Ag(111) resulted in a CLS of −2.1 eV. The N 1s BE for NO adsorbed on a p(4×4)-Ag(111) has been measured to be 402.1 eV,^26^ resulting in a value of 400 eV for the N 1s BE of atomic N in the bulk of Ag(111). As before, the O 1s BE was calculated with respect to O ion in the bulk, and referenced to the oxygen overlayer on the p(4×4) reconstruction. The calculated N 1s and O 1s for the adsorbates in Figure 7 are presented in Figure 8. For NO_2_ on the p(4×4) reconstruction, we calculated both the N 1s and O 1s CLS for both the NO_2_ adsorbed in the Ag vacancy and NO_2_ adsorbed on the O sites [NO_2_–O_site_].

**Figure 8:**
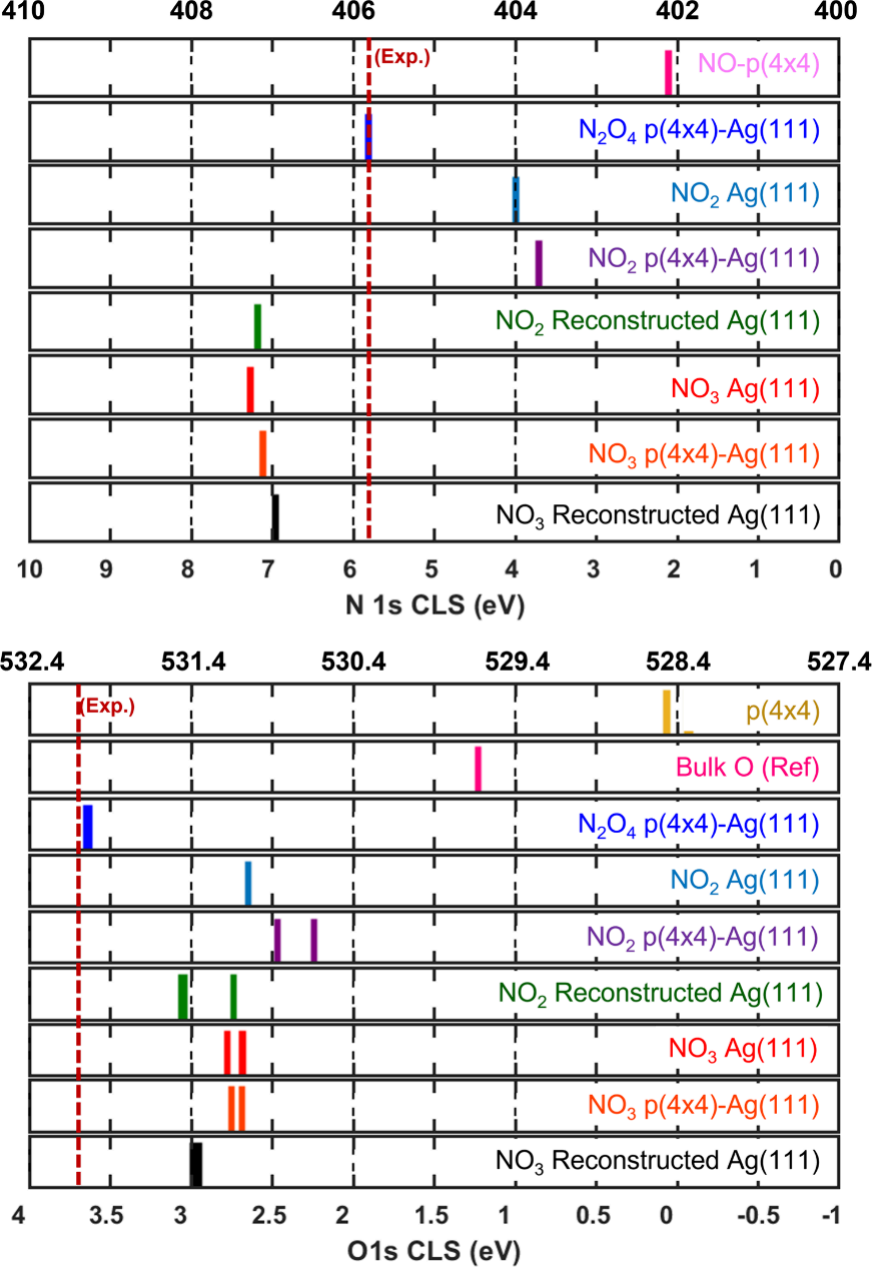
Calculated N 1s (top) and O 1s (bottom) core-level shifts for adsorbed N_*x*_O_*x*_ on pristine Ag(111) and p(4×4)-Ag(111). The bottom axis shows the relative shift with respect to the reference. The top axis shows the calculated absolute energies referenced to the known experimental values, i.e., N in adsorbed NO on p(4×4)-Ag(111) and O in pristine p(4×4)-Ag(111). The experimentally observed energy for each core–electron is shown with a red vertical dashed line.

The calculated N 1s BEs are shown in the top panel of Figure 8. Here, we find that NO_3_ and NO_2_ show a small spread of core–electron energies regardless of surface oxidation, with a calculated N 1s BE of 407.3–406.9 and 404.0–403.7 eV, respectively. Interestingly, NO_2_ adsorbed on the O site of the p(4×4) reconstruction exhibits a N 1s BE of 407.2 eV, suggesting that NO_2_ transforms chemically to a NO_3_ upon adsorption. The N 1s BE for N_2_O_4_ on the p(4×4) reconstruction is calculated to be 405.8 eV, in excellent agreement with our experimental observation. Our results for the N 1s BE of NO_2_ and NO_3_, centered respectively around 404 and 407 eV, agree with other experimental measurements and theoretical calculations.^4,6,26^

The O 1s CLS for the same adsorbates is presented in the bottom panel of Figure 8. Here, we find the spread of core–electron binding energies to be larger than for the N 1s, and more sensitive to surface oxidation. We calculate the O 1s BE for NO_2_ and NO_3_ on the pristine Ag(111) to be 531.0 and 531.1 eV, respectively. On the oxidized surface, the O 1s BE for NO_2_ [NO_2_–O_site_] is calculated to be 530.9 and 530.6 eV [531.5 and 531.1 eV], while for NO_3_, we find it to be 531.2 and 531.1 eV. The NO_3_-reconstructed structure shows the smallest energy spread, with an O 1s BE centered at 531.3 eV (±0.1 eV). Finally, the O 1s BE for N_2_O_4_ is calculated to be 532.0 eV, in close agreement with our experimental observation (see Figure 2). These results suggest that the adsorbed species observed in Figure 2 is not NO_3_, as repeatedly assigned in the past, but rather N_2_O_4_.

### Hydrogen Effect on N_2_O_4_ and NO_3_ Adsorption

We then analyzed the effect of hydrogen coverage on the N 1s and O 1s CLS for adsorbed species N_2_O_4_ and NO_3_. Namely, we considered adsorption of these two molecules on the *y*H/p­(4×4) reconstruction (*y* = 2, 4, and 6). The adsorption of NO_3_ was considered over the metallic ions of the p(4×4) reconstruction. This is reasonable since the N 1s BEs for NO_3_ all center around 407 ± 0.2 eV, regardless of surface oxidation or structure coordination. The adsorption energies of NO_3_ on the H-covered surfaces are found to be −2.54 and −2.68 eV for 2 H and 4 H coverages, respectively, whereas the adsorption energies for N_2_O_4_ are calculated to be −0.38 and −0.52 eV, respectively. The adsorption energies on the 6 H-covered surface are not presented, given the instability of the hydroxylated surface upon hydrogen adsorption (see Figure 5). The structures for the preferred configurations are shown in Figure S7. The effect of hydrogen coverage on the calculated N 1s and O 1s CLS for N_2_O_4_ is presented in Figure 9.

**Figure 9:**
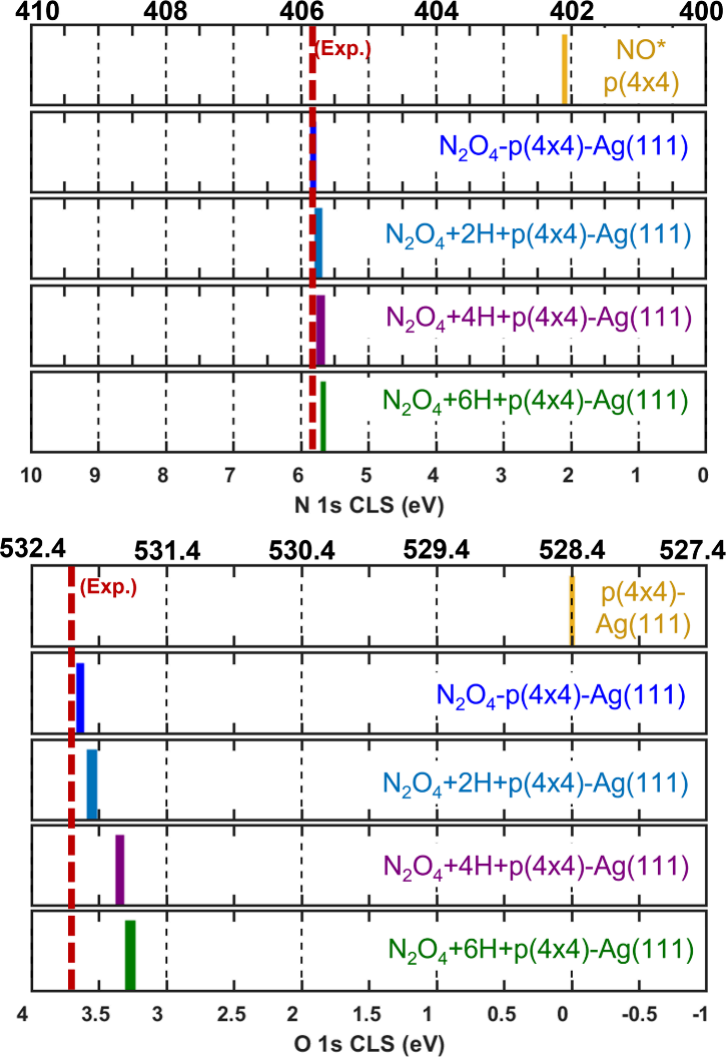
Calculated N 1s (top) and O 1s (bottom) core-level shifts for adsorbed N_2_O_4_ at different hydrogen coverages on p(4×4)-Ag(111). The bottom axis shows the relative shift with respect to the reference. The top axis shows the calculated absolute energies referenced to the known experimental values, i.e., N in adsorbed NO on p(4×4)-Ag(111) and O in pristine p(4×4)-Ag(111). The experimentally observed energy for each core–electron is shown with a red vertical dashed line.

For NO_3_, we calculate the N 1s BEs to be 407.2, 407.3, and 407.1 eV when adsorbed on the 2 H-, 4 H-, and 6 H-covered surfaces, respectively. On the same surfaces, we found the O 1s BE for NO_3_ to be centered at 531.2, 531.3, and 531.4 eV. The effect of surface hydroxylation on the N 1s BE of N_2_O_4_ was found to be almost negligible, with a N 1s BE centered around 405.7 eV for the 2, 4, and 6 H-covered p(4×4) reconstruction. However, the hydroxylation effect was more pronounced on the O 1s CLS of N_2_O_4_, where we calculated a shift to lower binding energies with increasing hydroxyl coverage on the surface. Specifically, we calculated the O 1s BE of N_2_O_4_ on the 2, 4, and 6 H/p(4×4) reconstruction to be 532.0, 531.8, and 531.7 eV, respectively. This is a shift of up to 0.4 eV on the O 1s BE with respect to the N_2_O_4_ adsorbed on the clean p(4×4)-reconstruction (O 1s BE = 532.0 eV). These results suggest that the O 1s binding energy of N_2_O_4_ can be affected by other species on the surface through long-range electrostatic interactions, while the N 1s BE should remain roughly unaffected.

To elucidate the electronic adsorption mechanism for N_2_O_4_, we interrogated the electronic structure of the N_2_O_4_ adsorbate on the pristine, and hydrogen-covered p(4×4) reconstruction. The projected density of states (PDOS) of the topmost layer for the surface, adsorbate/surface, and adsorbate/hydrogen-covered surface is shown in Figure 10. In the top panel of the Figure, we observe the PDOS on the p(4×4) reconstruction. Here, we find the O 2s states of the surface located at an energy between 17 and 16 eV below the Fermi level, while the O 2p states are found spreading from the valence band, 7 eV below the Fermi level, to the conduction band, above the Fermi level. The Ag 4d states are also found in the close proximity of the Fermi energy, overlapping with the O 2p state in the entire energy spread. Similarly, the Ag 5s states are found in the same energy spread as the Ag 4d and O 2p states. The sd states of the Ag ion right below the silver vacancy in the reconstructed surface are also shown. Upon adsorption of N_2_O_4_, middle panel in Figure 10, we note that there is little overlap between the sp states of the adsorbate and projected states of the oxidized surface. However, there is a slight decrease in the calculated intensity of the Ag 4d states at an energy of −2.9 eV, suggesting that there is indeed a weak chemical interaction between the surface and the adsorbate. We note that there is no distinct overlap between the subsurface Ag-ion-projected orbitals and the adsorbate. Upon hydroxylation of the surface, bottom panel in Figure 10, we note that there is a distinct rehybridization of the Ag 5s and 4d states with the N_2_O_4_ sp states at an energy of −7 eV, exactly where some H 1s states appear upon adsorption. This rehybridization correlates well with the effect of hydroxylation on the calculated O 1s and N 1s CLS for N_2_O_4_.

**Figure 10:**
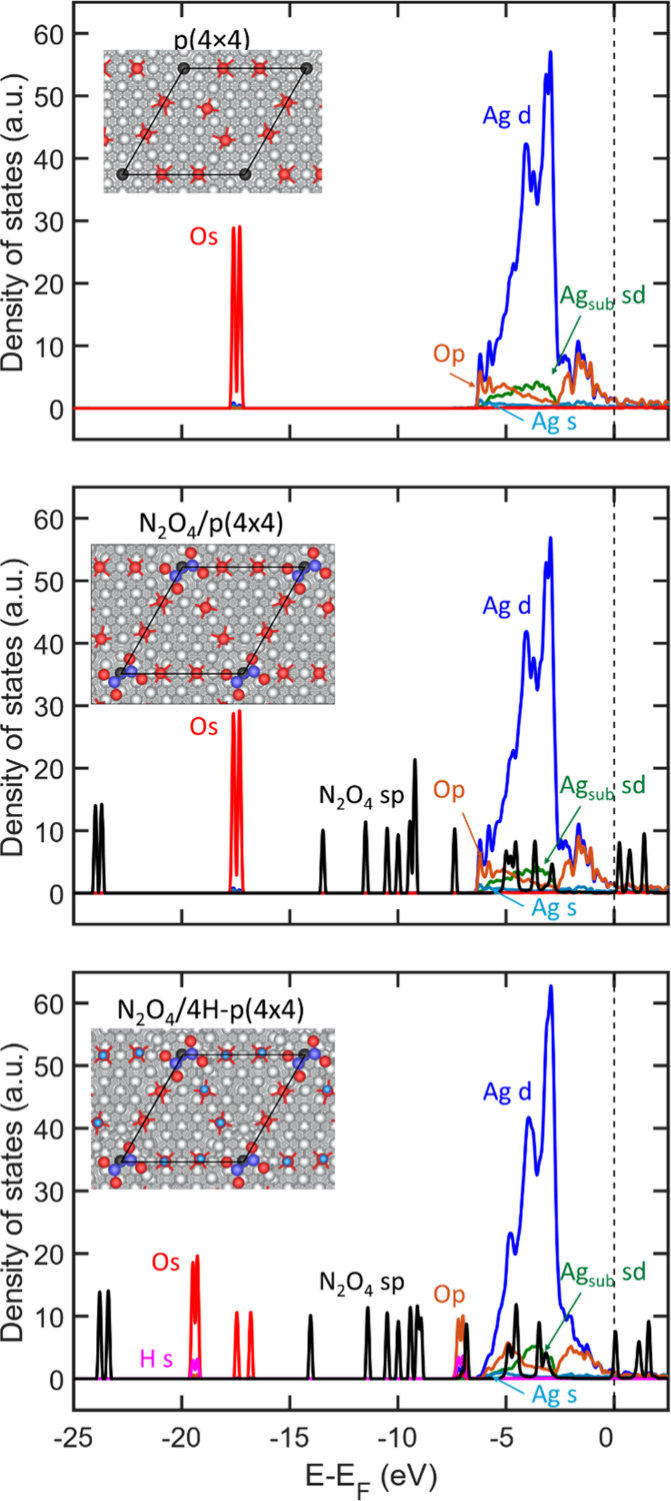
Calculated projected density of states for p(4×4)-Ag(111) (top), N_2_O_4_/p­(4×4)-Ag­(111) (middle), and N_2_O_4_/4H-p­(4×4)-Ag­(111) (bottom). The projected s and p states of the N_2_O_4_ molecule are summed together and shown in black. The sum of projected d and s states of Ag subvacancy ion is shown in green. Color code of projected states: Ag d (blue), Ag s (teal), O s (red), O p (orange), H s (magenta). Insets in the figures show the corresponding structures. Atomic color code: Ag (silver), Ag subvacancy (black), O (red), H (light blue).

A charge density difference analysis was also performed for N_2_O_4_ adsorbed on the p(4×4) reconstruction (see Figure 11). We plotted the charge density difference from the total system minus its individual components. We find that there is indeed a charge transfer from the Ag ions in the rim of the vacancy to the N_2_O_4_ adsorbate, showing that there is a weak interaction between the surface and adsorbate. This kind of interaction is consistent with the interatomic separation in the *z*-direction between the surface Ag and the molecule (2.48 Å). The adsorption energy, −0.54 eV, is slightly higher than what a simple physisorption would entail, suggesting there is perhaps a weak chemical exchange between the surface and N_2_O_4_.

**Figure 11:**
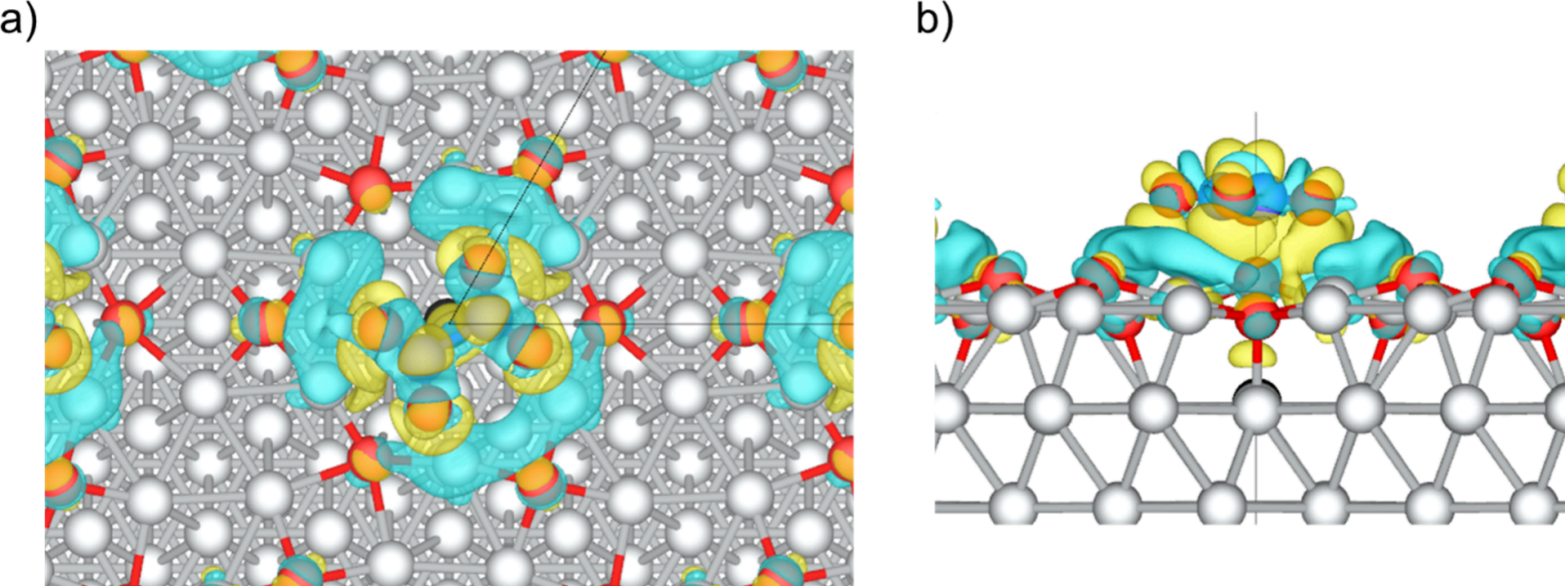
Charge density difference plot for N_2_O_4_ adsorption on p(4×4)-Ag(111). (a) Top view and (b) side view. Charge density difference is calculated as the difference of the charge density of the total system minus the pristine surface and the gas-phase N_2_O_4_ molecule. The yellow iso-surface indicates charge localization while the blue shows charge density depletion. Isosurfaces are plotted at an isovalue of ±0.0002 e/Å^3^. The corner of the surface unit cell is shown with black lines. A ball and stick model is used to show the atomic structures. Atomic color code as in Figure 7.

### Effect of Higher N_2_O_4_ Coverage on CLS

We evaluated the effect of N_2_O_4_ coverage on the calculated core–electron binding energies. The magnitude of the O 1s peak observed at 532.0 eV in Figure 2 is approximately twice as large as that of the surface oxygen species at 530.4 eV. This means there are approximately twice as many adsorbate oxygen ions than surface oxygen ions in the sample. Hence, we determined the adsorption configuration of N_2_O_4_ for the situation of having two and three molecules on the clean p(4×4) reconstruction (see Figure S8). Given there are six surface oxygens on the p(4×4) reconstruction, having a coverage of three N_2_O_4_ on the surface cell should give a comparable magnitude (2:1) of the observed adsorbate/surface oxygen O 1s peaks in Figure 2. Remarkably, the differential adsorption energies for two and three N_2_O_4_ are calculated to be −0.72 and −0.73 eV, respectively. Compared to the adsorption energy of −0.54 eV we calculated for the first N_2_O_4_ molecule to be adsorbed on this surface unit cell, we conclude that a higher N_2_O_4_ coverage might reflect the experimentally observed coverage. The calculated N 1s and O 1s CLS for increasing N_2_O_4_ coverage is shown in Figure 12.

**Figure 12:**
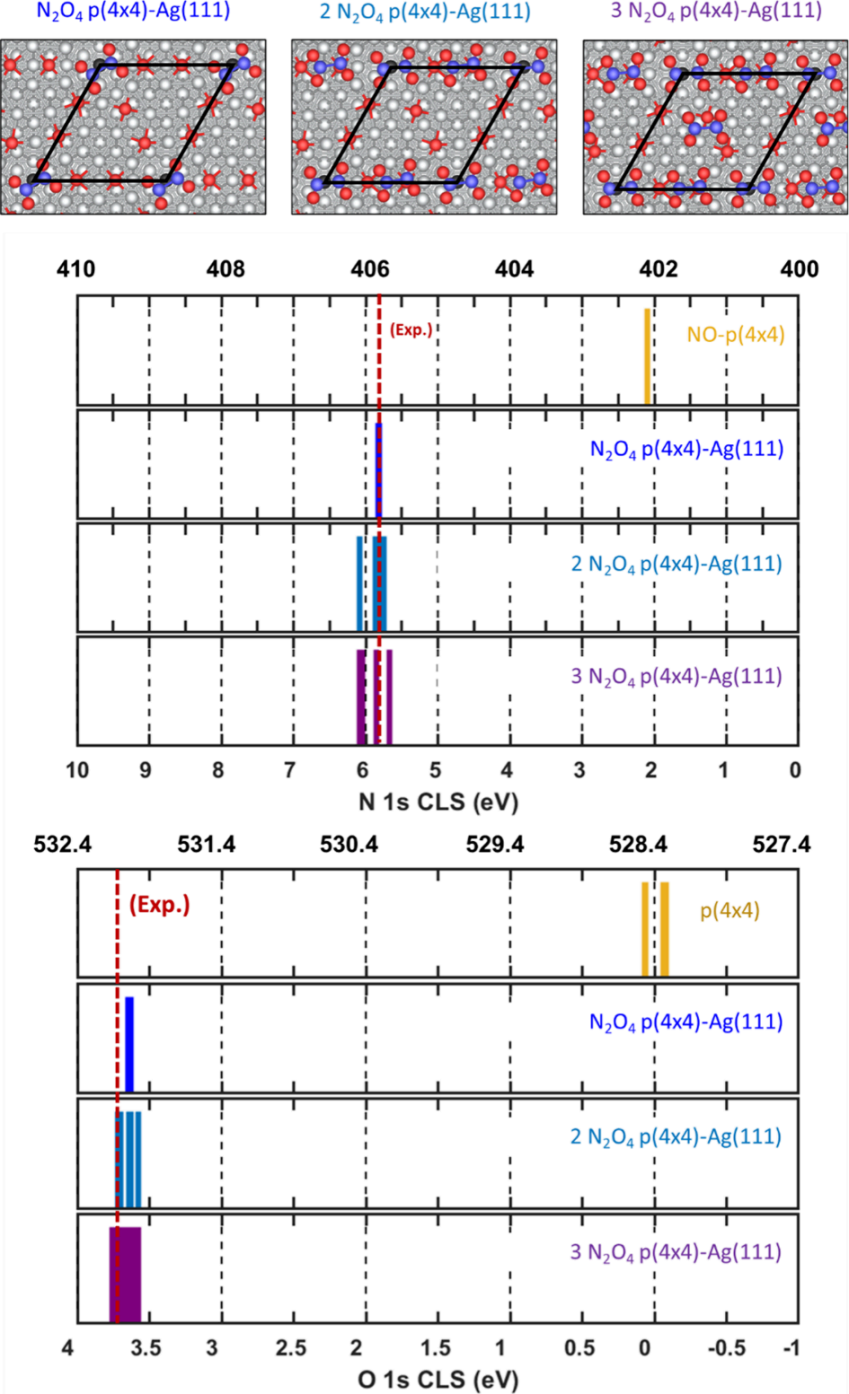
Calculated N 1s (top) and O 1s (bottom) core-level shifts for adsorbed N_2_O_4_ at different N_2_O_4_ coverages on pristine p(4×4)-Ag(111). The bottom axis shows the relative shift with respect to the reference. The top axis shows the calculated absolute energies referenced to the known experimental values, i.e., N in adsorbed NO on p(4×4)-Ag(111) and O in pristine p(4×4)-Ag(111). The experimentally observed energy for each core–electron is shown as a red dashed line. Structures for the adsorbates are shown in the topmost panel. Atomic color code as in Figure 7. A ball-and-stick model is used to show the atomic structures.

The N 1s BE is presented in the top panel of Figure 12. We find that increasing the coverage to two N_2_O_4_ molecules in the unit cell induces a slight spread on the N 1s BE with the average value centered at 405.9 eV ± 0.2 eV. For the situation with three N_2_O_4_ molecules in the unit cell, we observe the same behavior and energies as for the case of two molecules. In a similar fashion, increasing the adsorbate coverage on the p(4×4) leads to a larger spread on the O 1s BEs too (bottom panel Figure 12). Namely, the calculated O 1s BE for two N_2_O_4_ molecules in the unit cell exhibits an average value of 532.0 eV, with a spread of ±0.1 eV, while three N_2_O_4_ molecules in the unit cell produces an average value of 532.1 eV, with the same spread in energy. These results suggest that a higher coverage of N_2_O_4_ only leads to a slightly broader peak, centered roughly at the same core–electron binding energies calculated at the lower coverage limit.

## Discussion

The N 1s BE peak at 405.8 eV on silver has been traditionally assigned to adsorbed NO_3_.^15,16^ However, our results suggest that this assignment should be reconsidered. The assignment of NO_3_ as the dominant adsorbate on silver has been partially based on IR measurements,^19^ where surface selection rules can conceal the presence of other adsorbates. X-ray photoelectron spectroscopy is a better-suited technique for fingerprinting adsorbates. Nevertheless, XPS spectra can be difficult to interpret and assign. The role of theoretical investigations, such as the one presented here, is to suggest adsorbates that are consistent with experimental observation. The fact that we calculate the N 1s BE for NO_3_ to be around 407 eV, in agreement with experimental observations on other systems,^6^ is strong evidence to support our argument. Additionally, the fact that we calculate N_2_O_4_ core–electron binding energies to be in good agreement with experimental measurements suggests that the adsorbate on the silver system is probably not NO_3_ but more likely N_2_O_4_. Furthermore, our calculations elucidate the assignment made in ref 16 for NO_2_ and NO_3_ to the same N 1s BE (405.8 eV) while having different O 1s BEs (530.4 and 531.8 eV, respectively). As we showed in this work, the presence of surface hydroxyl groups can shift the O 1s BE of N_2_O_4_ to lower binding energies, while barely affecting the position of the N 1s peak, suggesting that the O 1s BE shift observed upon increasing NO_2_ dosing in ref 15 could be the result of some surface-species build-up that interacts electrostatically with adsorbed N_2_O_4_.

To reconcile our findings with the existing experimental characterization for NO_2_ adsorption on silver, we obtained the experimental and theoretical IR spectra for our proposed adsorbates. We find that the experimental IR measurement fits the reported peaks for NO_2_ adsorption on Ag(111) within ±2 cm^*–*1^ (see Figure S9).^14,19^ However, none of our individually tested theoretical adsorbates produces the same number of peaks at the right wavenumber (see Figure S10). While a superposition of the different adsorption modes for NO_3_ can computationally produce the experimentally observed peaks within reasonable accuracy, the calculated IR spectra for N_2_O_4_ produces, at best, only some of the experimental peaks (see Figure S10). Nonetheless, one must note that the relative intensity of NO_3_ on Ag(111) is one order of magnitude larger than that calculated for N_2_O_4_ at the low coverage limit. In fact, the calculated IR intensity for NO_3_ at a step site is up to four orders of magnitude larger than that of N_2_O_4_ on the p(4×4) (see Figure S10). This extreme difference in calculated intensities for different adsorbates suggest that a minority amount of NO_3_ on the surface could dominate the IR spectra completely (see Figure S10), while barely changing the magnitude or position of the XPS peaks. This interpretation is also consistent with the observed decrease in the intensity of the surface oxygen O 1s peak in Figure 2 upon NO_2_ dosing, where a small amount of surface oxygen is lost or transformed upon adsorption of NO_2_, potentially to make residual NO_3_.

Another possible explanation for the IR and XPS calculations is that the adsorbate observed experimentally could be an isomeric form of N_2_O_4_.^73^ Indeed, the ONO-NO_2_ isomer has been assigned in the literature to the N 1s BE of 405.6 eV on a gold-stepped surface.^9^ We investigated the adsorption of this isomer on the p(4×4) reconstruction but failed to find a stable adsorbed configuration.

At first glance, one seemingly puzzling matter with the assignment to N_2_O_4_ is its relatively weak adsorption energy (−0.72 eV at higher coverage) in comparison to that of NO_3_ (−2.41 eV) on the p(4×4) reconstruction. From a thermodynamic perspective, there appears to be a significant driving force to form NO_3_. Moreover, high-vacuum conditions, like the ones used to perform XPS measurements, could potentially drive desorption of N_2_O_4_ from the silver surface. We have rationalized the presence of N_2_O_4_ instead of NO_3_ in terms of its reaction energy with respect to NO_2_(g) and O_2_(g). The adsorption of NO_2_ on the p(4×4), NO_2_ (g) + * → NO_2_*, has a reaction energy of −1.21 eV. From here, making NO_3_ on the surface requires additional atomic oxygen, NO_2_* + 1/2 O_2_ (g) → NO_3_*, only having a reaction energy of −1.22 eV. In fact, if we consider the direct adsorption of NO_2_ on an oxygen site of the p(4×4), [NO_2_–O_site_], NO_2_ (g) + O_site_ → NO_2_*O_site_, then the reaction energy is only −0.97 eV, while using the higher coverage energetics, we calculate the reaction energy to make N_2_O_4_ on the surface, 2 NO_2_ (g) → N_2_O_4_ (p), to be −1.65 eV. This means that if NO_3_ is not made in the gas phase, then the formation of NO_3_ on the p(4×4) is less exothermic than the formation of N_2_ *O*_4_(*p*). Although thermodynamic analysis suggests that N_2_O_4_ formation is more favorable over that of NO_3_ formation, we cannot exclude kinetic factors controlling either of these two processes.^26^ Explaining the existence of N_2_O_4_ in appreciable coverages under high-vacuum conditions reflecting our XPS measurements requires a more detailed thermodynamic analysis, which we address in the Supporting Information section “N_2_O_4_ under high-vacuum conditions”. Our thermodynamic analysis shows that N_2_O_4_ stability at high-vacuum conditions is possible at room temperature and is favored by high surface coverages. We evaluated the equilibrium surface coverage of N_2_O_4_ (Figure S12) and found that, even when employing the reaction energetics in the low-coverage limit (Δ*E*_rx_[2 NO_2_ (g) → N_2_O_4_*] = −1.65 eV), an equilibrium coverage of approximately 0.02 ML of N_2_O_4_ is predicted under high-vacuum conditions on the p(4×4) surface unit cell. Moreover, the stability of the different surface coverages upon NO_2_ adsorption, as monomer or dimer, was determined by calculating their surface free energy at *T* = 298 K as a function of the NO_2_ chemical potential with the use of ab initio thermodynamics (Figure S13). We find that under high-vacuum conditions, higher dimer surface coverage (three N_2_O_4_) is thermodynamically preferred over lower monomeric NO_2_ adsorption. This is understood from the reaction energy for the high coverage dimer configuration (Δ*E*_rx_[6NO_2_ (g) → 3N_2_O_4_*] = −4.76 eV).

One must note that errors in gas-phase energies have been previously reported for nitrogen-containing species^74^ and are common in carbon-based molecules.^75–80^ In fact, through temperature-programmed desorption experiments, the adsorption energy of NO on Ag(111) has been estimated to be −1.08 eV,^81^ whereas we calculate that quantity to be −0.64 eV, suggesting that our calculated adsorption energies are underpredicted. Nevertheless, even if our gas-phase energies are overpredicted, and even if our calculated energies for nitrogen-containing species are off by *∼*0.3 eV, our conclusions are unaffected. Our findings support the assignment of OH groups to the O 1s BE of 530.4 eV and suggest that there is a need to revisit the experimental assignment of NO_3_ to the N 1s BE of 405.8 eV on silver surfaces, as N_2_O_4_ appears to be a more suitable assignment.

## Conclusions

We have used density functional theory calculations, in combination with X-ray photoelectron spectroscopy measurements, to investigate the adsorption of H_2_ and NO_2_ on oxygen precovered silver surfaces, dominated by Ag(111). The thermodynamic stability of different oxygen covered Ag(111) was addressed to determine a reasonable model for the state of the oxidized surface. The p(4×4) reconstruction was determined to be a reasonable model to study the adsorption of other molecules on oxidized Ag(111). The formation of surface hydroxyl groups on the p(4×4) reconstruction of Ag(111) was investigated and characterized with core-level shift (CLS) calculations. Formation of OH groups is found to be both thermodynamically and kinetically feasible at room temperature. The O 1s CLS of surface hydroxyl groups explains the oxygen-surface features on oxygen-modified Ag(111) and offers a possible solution to the debate on the origin of the XPS peak at 530.0–530.7 eV.

The adsorption of NO_2_ in dimer form (N_2_O_4_) on the p(4×4) reconstruction elucidates the N 1s BE signature observed at 405.8 eV, whereas the N 1s BE signature for NO_3_ is predicted to be closer to 407 eV. The presence of surface hydroxyl groups induces a shift in the O 1s CLS of N_2_O_4_ to lower binding energies and provides an explanation for the experimentally observed shift reported in the literature (531.8 to 531.4 eV) at the same N 1s BE (405.8 eV) [state IV and state V].^15,16^ A higher coverage of N_2_O_4_ is predicted to further stabilize the intermediate and leads only to a slightly broader signature on the core–electron binding energies.

The present work suggests that the species assignment for N 1s BE of 405.8 eV on oxygen-modified silver surfaces should be reconsidered and provides fundamental insights for the future development of silver-based NO_2_ removal and monitoring technologies.

## Supplementary Material





## Notes

Structural models, experimental IR, and simulated IR (PDF)
Molecular structures (ZIP)
