# Supplementary material for: NO2 Adsorption on Oxygen-Modified Ag at Ambient Conditions
Source: J Am Chem Soc. 2025 Nov 5;147(46):43139–52. doi: 10.1021/jacs.5c16683 (PMC12636020; doi:10.1021/jacs.5c16683)
Supplement: Supplementary file 1 [file ja5c16683_si_001.pdf]

Supporting Information:
NO_2 adsorption on oxygen-modified Ag at ambient conditions

Alvaro Posada-Borbón*,† Trenton Wolter,† Huaizhe Yu,‡ Evangelos Smith,† James J. Schauer,† Reid C. Van Lehn,† Victor M. Zavala,† Nicholas L. Abbott*,‡ and Manos Mavrikakis*,†

†Department of Chemical and Biological Engineering, University of Wisconsin-Madison, WI 53706, United States

‡Robert Frederick Smith School of Chemical and Biomolecular Engineering, Cornell University, 1 Ho Plaza, Ithaca, New York 14853, USA

E-mail: palvaro@chalmers.se; nabbott@cornell.edu; emavrikakis@wisc.edu

Cleaning of glass substrates for gold deposition.

Glass microscope slides were cleaned according to published procedures using an acidic piranha solution [70: 30 (% v/v) H_2SO_4 (70%): H_2O_2 (30%)].[S1] Piranha is extremely corrosive and potentially explosive, and should not be exposed to organic material under any circumstance. Briefly, the glass slides were immersed in a piranha bath at 60-80 °C for at least 1 hour and then rinsed in running deionized water for 2-3 mins. The slides were subsequently immersed in basic piranha solution [70: 30 (%v/v) KOH (45%): H_2O_2 (30%)] and heated to between 60 and 80 °C for at least 1 h. Finally, the slides were rinsed sequentially in deionized water and ethanol and then dried under a stream of nitrogen. The clean slides were stored in a vacuum oven at 110 °C overnight. All other glassware was cleaned before use.

XPS measurements.

X-ray photoelectron emission spectra of the Ag/Au surface using a thick Ag overlayer (25 ML of Ag) were also collected upon exposure to 10 ppm of NO_2 (balanced by N_2 at 1 atm) at a temperature of 298 K. The observed spectral peaks for O 1s and N 1s core-electron binding energies are found to be the same as for the thinner Ag overlayers (1.1 ML of Ag) reported in the main manuscript (see Figure S1).

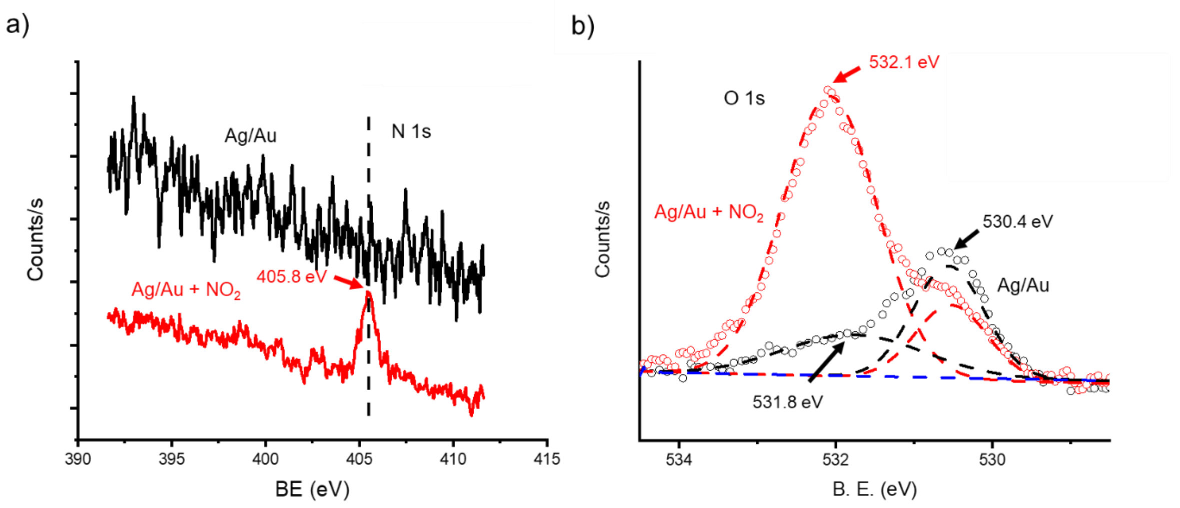

Figure S1: Photoelectron emission spectra of the Ag/Au surface before (black) and after (red) exposure to 10 ppm of NO_2 (balanced by N_2 at 1 atm) at a temperature of 298 K on a 25 ML Ag/Au system. **a**) N 1s spectra. **b**) O 1s spectra. Decomposed peaks are shown as dashed lines.

O 1s Binding energies: 1 ML Ag/Au vs 25 ML Ag/Au.

A question that arises with the 1 ML Ag/Au system is whether the growth of the Ag on the Au films is truly 2D or whether some multilayers (3D) islands of Ag form prior to a contiguous overlayer of Ag forming on the Au film. This question is particularly relevant because the O 1s BE for the oxygen surface species on the 1 ML Ag/Au and the 25 ML Ag/Au have the same peak position at 530.4 eV, with different broadness. It is reasonable to assume that for the 25 ML Ag/Au, dislocations may have formed, and the native lattice constant of Ag would have been regained (as opposed to retaining the underlying Au lattice constant). Thus, the O 1s BE on the 25 ML Ag/Au would essentially be identical to that on pure Ag, while the effect of the Au substrate on the 1 ML Ag/Au system is, *a priori*, unclear.

Based on studies of other Ag/metal systems, it might be expected that there would be a difference between XPS adsorbate BEs for one ML of Ag versus 25 ML of Ag. The difference in the XPS BEs could potentially result from strain induced by a lattice mismatch between the two metals and electronic hybridization at the Ag/Au interface. These two effects would effectively lead to a change in the electrostatic potential generated at the metallic surface, affecting how the core electrons of an adsorbate are perturbed upon adsorption. To understand if it is possible for us to determine whether a single monolayer of Ag or an island of Ag is formed, and to understand why both the 1ML and the 25 ML Ag/Au systems exhibit the same O 1s BE for surface oxygen, we need to understand, 1) the effect of

lattice mismatch and 2) the effect of electronic hybridization at the Ag/Au interface.

1) The lattice mismatch between Au (lc=4.078 Å) and Ag (lc=4.086 Å) is minimal (~0.2%). Hence, the impact on core-electron binding energies is likely comparable to that of natural defects in both the 1 ML and 30 ML systems. Given that neither surface is a pristine single crystal, some level of defects is expected, suggesting that the lattice mismatch effect on binding energies is similarly modest.

2) The magnitude of electronic hybridization at the Ag/Au interface and its effect on the core-electron binding energies can be understood with the use of DFT calculations and the d-band model.[S2]

On transition metals where the d-band model applies, there is a linear correlation between the position of the d-band center of a system and the induced core-level shift (CLS) on an adsorbate.[S3] This is because both shifts in the d-band center as well as shifts on the core electron eigenvalues are mostly the result of the electrostatic potential.[S3] Hence, as long as the d-band center of the Ag/Au system is similar to the d-band center of Ag, the induced change in the core-electrons BE of the adsorbate can be expected to be small. The d-band center of 1 ML of Ag(111) on Au(111) was calculated in Ref. [S4] to be -4.06 eV, while the d-band center of Ag(111) was calculated to be -4.15 eV. [S4] This difference, 0.09 eV, is small enough for one to assume the induced change in the O 1s CLS should be of a similar magnitude. In fact, using the adsorption energy as a proxy for the CLS (given the linear relation between the two), we calculate the adsorption energy of O on the fcc site on both surfaces. We found the O adsorption energy on a 1 ML Ag(111)/Au(111) to be Eb [O/1ML] = -0.70 eV, comparable to the calculated value on pristine Ag(111) Eb [O/Ag(111)] = -0.64 eV), suggesting indeed there is a small effect induced by the electronic hybridization at the interface for the 1 ML Ag/Au system, in comparison to the pristine system.

From the results in the literature for the d-band center and the calculated adsorption energies on the 1ML Ag(111)/Au(111) and the Ag(111), we conclude that the electronic properties of this particular system cause the core-electron BEs on the 1ML Ag(111)/Au(111) and the 25 ML Ag(111)/Au(111) to be similar. Although our calculations do not rule out the possibility of 3D island formation, they do not provide evidence for 3D island formation, consistent with prior experimental conclusions cited in the main text. [Ref. [30]- Ref. [32]]

Surface cell p(4×4)-Ag(111).

The oxidation of Ag(111) was investigated considering different oxygen coverages on the surface. The lower oxygen coverages (0 to 0.16 ML) were interrogated on a 4-layers thick Ag(111) on a (4×4) supercell, while surface reconstruction at 0.5 ML was considered on the p(4×4)-Ag(111) surface structure, described with a total of 5 layers, also on a (4×4) surface cell (Figure S2). Higher oxygen coverage, emulating the initial formation of $Ag_2O(111)$ was studied on the p(7×7)-reconstruction (Figure S3).

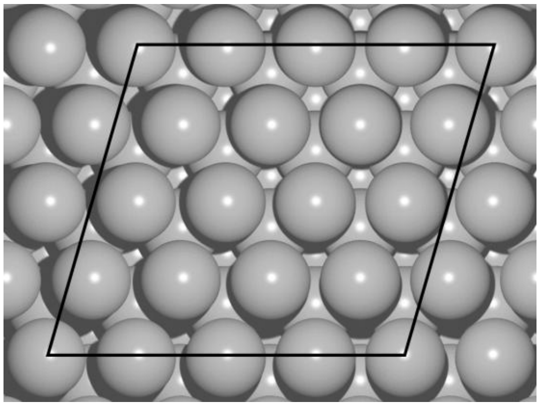

Figure S2: Top view of surface cell model for p(4×4)-Ag(111). Atomic color code: Ag (Gray). Surface unit cell is show in black lines.

p(7×7)-Ag(111) reconstruction.

The p(7×7)-reconstruction is described as the adsorption of two layers of p(3×3)-$Ag_2O(111)$, supported on a four-layered p(7×7)-Ag(111) substrate. The stability of the p(7×7)-Ag(111) structure was probed by optimizing the position and stacking of the Ag_2O overlayer, as reported in Ref.[[26]], over the metallic p(7×7)-Ag(111). The p(7×7)-reconstruction was optimized from the reported structure by displacing the Ag_2O overlayer over the xy-plane with respect to the Ag(111) substrate. Additionally, two cases were considered where the degree of oxidation of the overlayer is different. One case is when the adsorbed overlayers of Ag_2O are fully stoichiometric ($Ag_{72}O_{36}$), i.e, when the two adsorbed layers are composed of $Ag_2O(111)$ tri-layers with the proper Ag/O ratio, hence bonding with the Ag(111) substrate through Ag-O and Ag-Ag bonding. The other case considered the situation where all the oxygen ions are removed from the bottom tri-layer on the bottom-most layer in the $Ag_2O(111)$ overlayer, non-stoichiometric ($Ag_{72}O_{27}$), hence adsorbing on the Ag(111) trough Ag-Ag bonding. Both surfaces were originally adsorbed following the stacking of the Ag(111), as reported in Ref. [[26]], so the Ag-ion on the bottom layer of the overlayer would

resemble a slightly reconstructed and oxidized Ag(111) layer. A total of 16 structures were analyzed for each of the stoichiometric and non-stoichiometric surfaces, corresponding to overlayer displacements in increments of 0.5 Å in the x and y coordinates (across a total of 2 Å in each lattice direction). The optimal structure for the stoichiometric configuration was found to be 0.7 eV more stable than the original structure, whereas the non-stoichiometric configuration was found to be 2 eV more stable than its original structure. The structure files for the optimal configurations are provided as .cif files separately. The energetically preferred structures for stoichiometric and non-stoichiometric p(7×7)-Ag(111) reconstructed surfaces are shown in Figure S3.

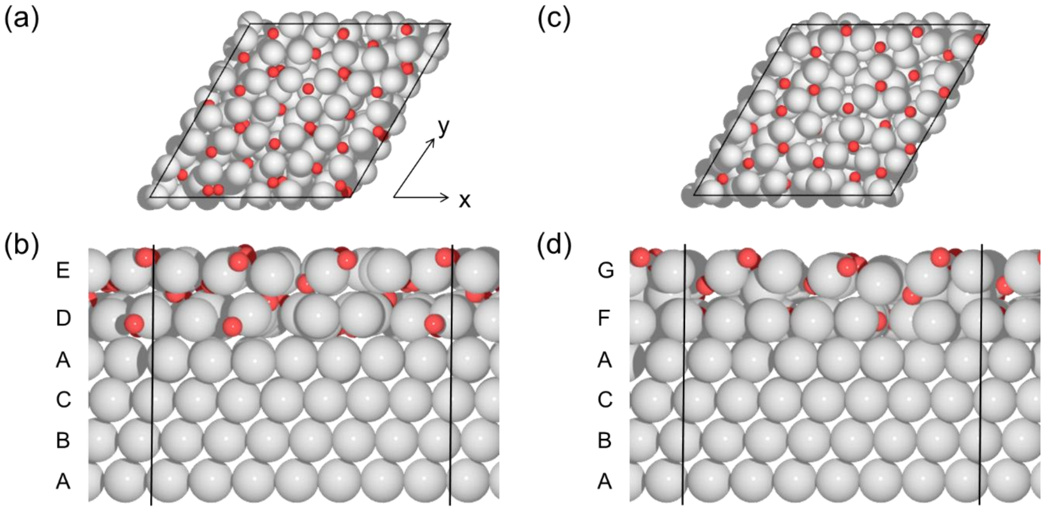

Figure S3: Structural models for stoichiometric and non-stoichiometric p(7×7)-Ag(111) re- construction. **a**) Top and **b**) cross-sectional views of stoichiometric $Ag_{72}O_{36}$/Ag(111). **c**) Top and **d**) cross-sectional views of non-stoichiometric surface $Ag_{72}O_{27}$/Ag(111). Layer stacking is denoted to the left of the slab cross-sectional view. Atomic color code: Ag (Gray), O (red). Surface unit cell is shown in black lines.

N_xO_y adsorption on p(4×4)-Ag(111).

The adsorption of N_2O, N_2O_3, and N_2O_5 was investigated on the oxidized p(4×4)-reconstruction. The structural models for the most stable states of the adsorbed molecules are shown in Figure S4. The calculated N 1s CLS for the structural models in Figure S4 suggest that the adsorbed N_2O_3, and N_2O_5 decompose to NO, NO_2, and NO_3 on the surface. The calculated N 1s CLS are shown in Figure S5.

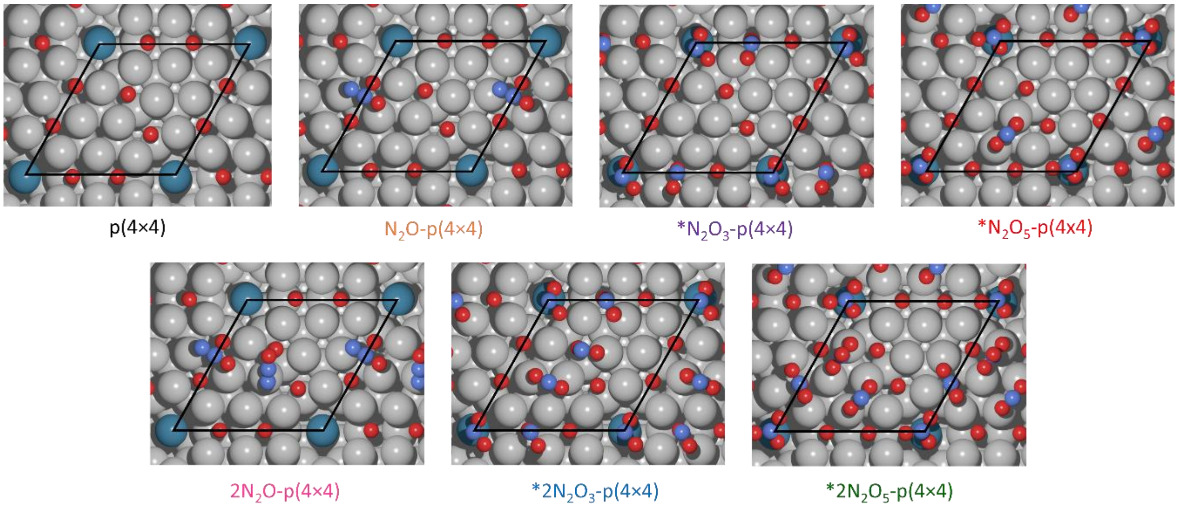

Figure S4: Minimum-energy structural models for N_2O, N_2O_3, and N_2O_5 adsorbed on the p(4×4)-Ag(111) reconstruction. Atomic color code: Ag (Gray), Sub-Ag-vacancy site (Dark blue), O (red), N (blue). Surface unit cell is shown in black lines. '*' Denotes dissociative adsorption. N_2O_3 and $2N_2O_3$ dissociate into $NO_{(phys)}$ and NO_2, whereas N_2O_5 and $2N_2O_5$ dissociate into NO_2 and NO_3.

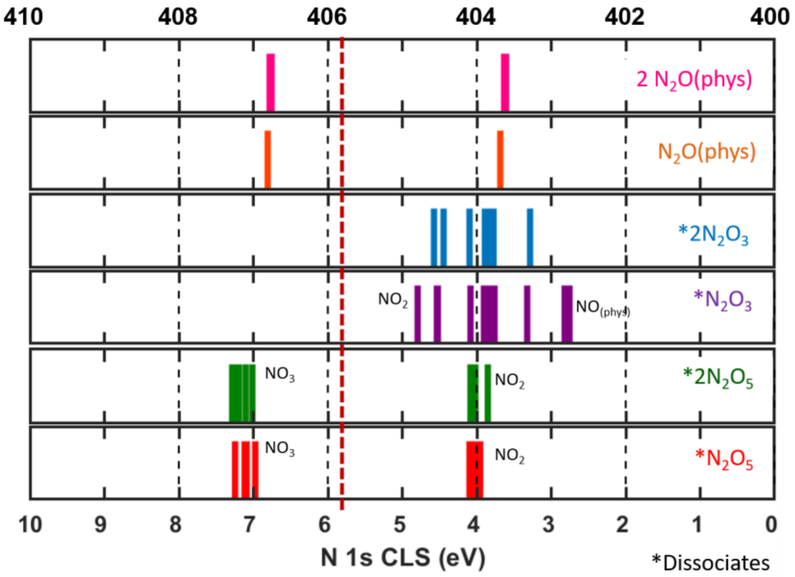

Figure S5: Calculated N 1s core-level shifts for minimum energy structures for adsorbed N_2O, N_2O_3, and N_2O_5 on the p(4×4)- Ag(111) reconstruction. Bottom axis shows the relative shift with respect to the NO reference (402.1 eV). Ref.[26] Top axis shows the calculated absolute energies referenced to the known experimental values, i.e., N in adsorbed NO on the p(4x4)-Ag(111) reconstruction.[26] Experimentally observed energy for each core-electron is shown as red dashed line (405.8 eV).

Hydrogen adsorption on the p(4×4)-Ag(111) reconstruction.

The adsorption H_2 on the p(4×4) Ag(111) reconstruction was investigated through the heterolytically dissociative adsorption step: $H_2(g) \rightarrow OH^* + H^*$. This type of mechanism has been found to be the

preferred path for H_2 dissociation on oxidized surfaces.[S5] Our calculated potential energy diagram and transition state (TS) are shown in Figure S6. For reference, we calculate the H-H bond energy to be −4.54 eV, in excellent agreement to the experimentally known value of −4.52 eV.

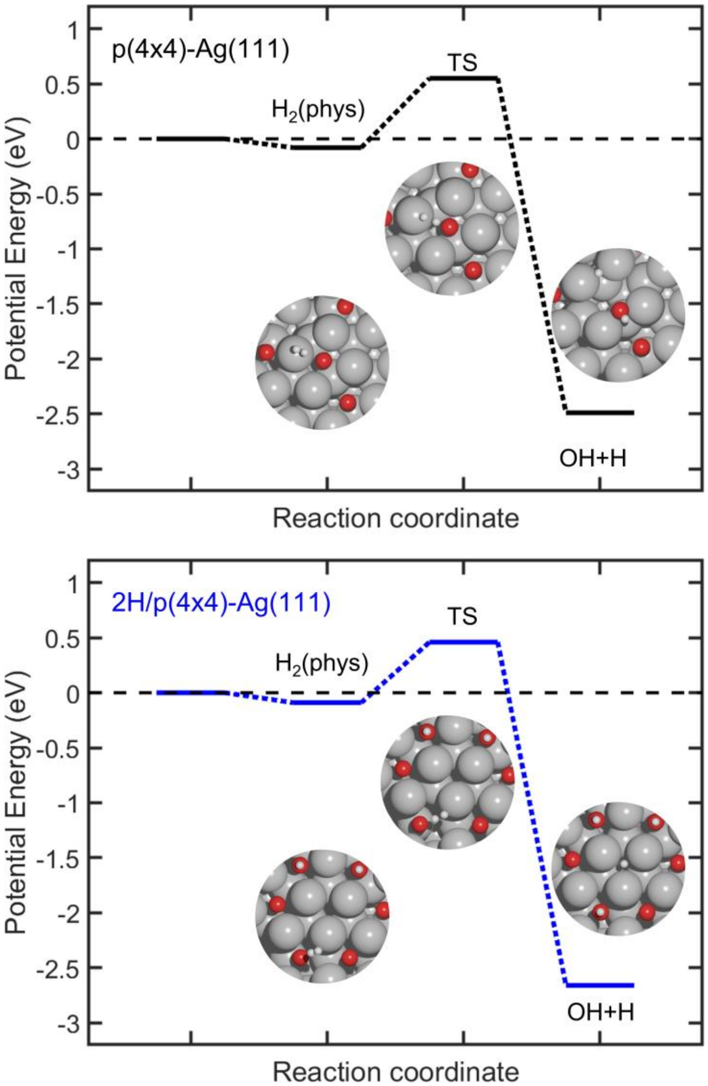

Figure S6: Potential energy diagram from $H_2(g) \rightarrow OH^* + H^*$ on (**top**) a pristine p(4×4) Ag(111) reconstruction, and (**bottom**) a 2H/p(4×4)-Ag(111) reconstruction.

The effect of surface hydroxyl groups on the CLS for N_2O_4 adsorbed on the p(4x4) Ag(111) reconstruction was evaluated at a H pre-coverage of 2H, 4H, and 6H in the unit cell. The structural models of the evaluated cases are presented in Figure S7. The effect of hydroxyl on the CLS for N_2O_4 on the p(4x4)-Ag(111) reconstruction is discussed in the main text.

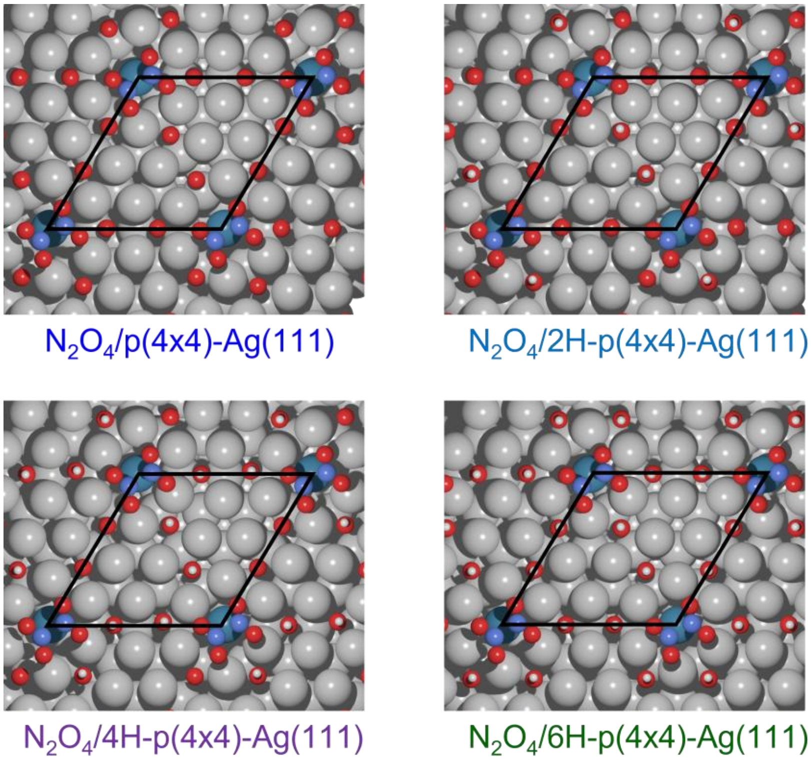

Figure S7: Calculated structural models for N_2O_4 adsorption on the pristine, 2 H, 4 H and 6 H covered p(4×4)-Ag(111) reconstruction. Ag-vacancy site is highlighted by showing the subsurface site in dark blue. Atomic color code: Ag (Gray), Sub-Ag-vacancy site (Dark blue), O (red), N (blue), H (white). Surface cell is shown in black lines.

N_2O_4 coverage on the p(4×4)-Ag(111) reconstruction.

The effect of N_2O_4 coverage was investigated on the p(4×4)-Ag(111) reconstruction. The minimum energy structural models for the adsorbed molecules are shown in Figure S8. The calculated N 1s and O 1s CLS for the structural models in Figure S8 are presented in the main text.

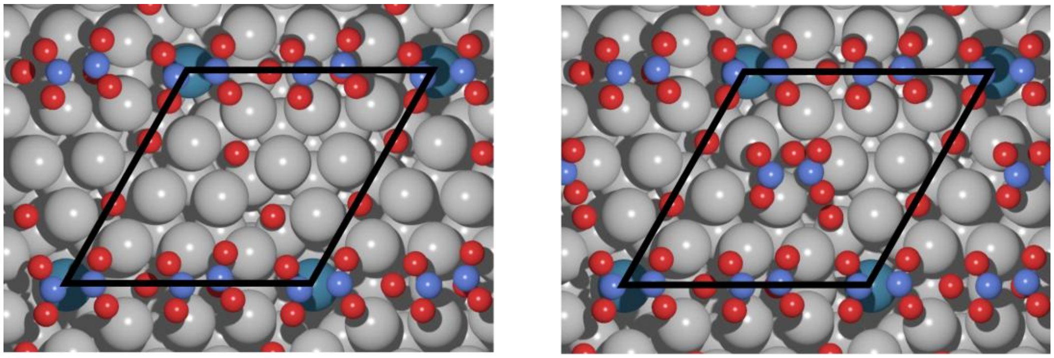

Figure S8: Calculated structural models for 2 N_2O_4 and 3 N_2O_4 molecules adsorption on the p(4×4)-Ag(111) reconstruction. Ag-vacancy site is highlighted by showing the subsurface Ag atom in dark blue. Atomic color code: Ag (Gray), Sub-Ag-vacancy site (Dark blue), O (red), N (blue).Surface unit cell is shown in black lines.

Fourier transformed polarization-modulation infrared reflectance absorbance spectroscopy (PM-IRRAS) of NO_2 adsorption on O-covered Ag(111).

For PM-IRRAS measurements, Au coated Si wafers were used to provide a reflecting substrate. 1.13 ML Ag was deposited on Au-coated Si wafers by underpotential deposition. IR spectra of 1.13 ML Ag deposited onto Au-coated silicon wafers pre and post NO_2 exposure were obtained using a Nicolet Magna-IR 860 FT-IR spectrometer, which employed a photoelastic modulator (PEM-90, Hinds Instruments, Hillsboro, OR), synchronous sampling demodulator (SSD-100, GWC Technologies, Madison, WI), and a liquid N_2-cooled mercury cadmium telluride (MCT) detector. All spectra (1000-4000 cm^{-1}) were recorded at an incident angle of 83° with the modulation centered at either 2200 cm^{-1} or 1500 cm^{-1}. For each sample, 1000 scans were taken at a resolution of 4 cm^{-1}. Data were collected as differential reflectance vs wavenumber, and the spectra were normalized and converted to absorbance units via the method outlined in Frey et al. NO_2 exposure was performed using 10 ppm NO_2 (balance nitrogen at 1 atm) at 298 K for 30 minutes in a gastight homemade IR chamber with sodium chloride windows that fit within the PM-IRRAS instrument.[S6, S7] The IR chamber had inlet and outlet valves that could be opened to allow NO_2 to flow into the chamber and then closed to place the chamber into the PM-IRRAS instrument.

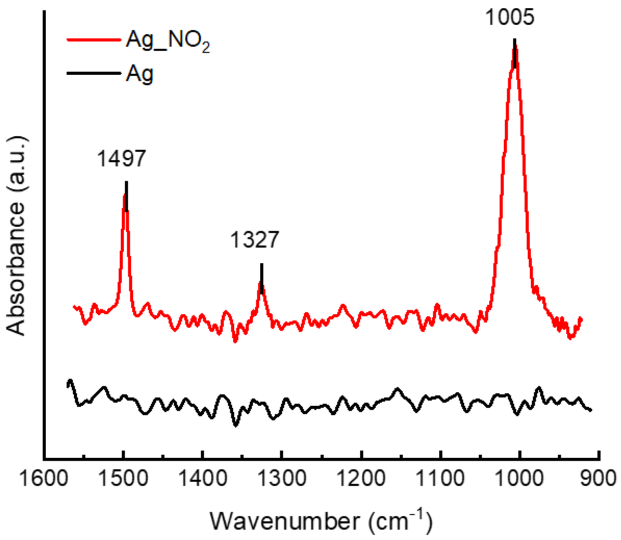

Figure S9: Observed IR spectra of 1.13 ML Ag on Au surface before and after NO_2 exposure.

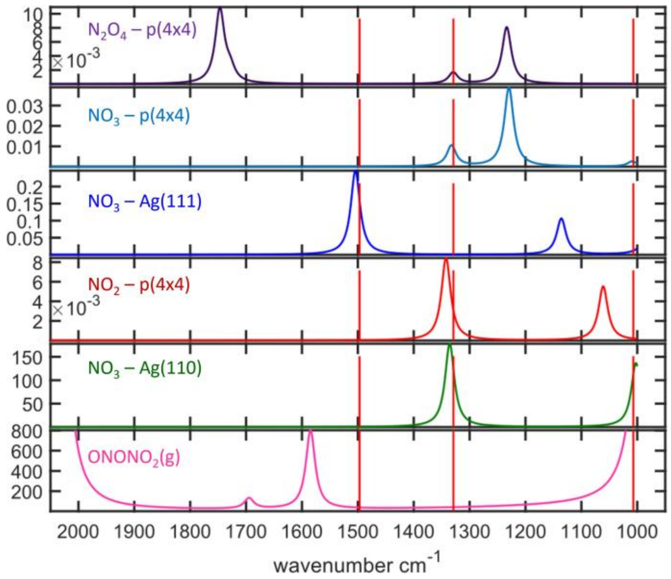

Figure S10: Calculated IR spectra for different adsorbed species on Ag surfaces. Experimentally observed peaks are shown with a red vertical line at the corresponding frequency.

N_2O_4 under high vacuum conditions

A key question related to the assignment of N_2O_4 to the N 1s BE of 405.8 eV is its presence on the surface under high vacuum conditions (reflecting our XPS measurements conditions), mainly because of its relatively weak calculated adsorption energy (-0.72 eV). However, given that the observed N_2O_4 is the result of a surface reaction on the silver-based surface, it is important to understand the stability of N_2O_4 on the surface from the N_2O_4 formation reaction's thermodynamics

and the reaction's reactants (NO_2(g)).

To demonstrate that an analysis of the energetics referenced to NO_2(g) as initial state is required, we calculated the equilibrium concentration for gas-phase N_2O_4 at high vacuum (UHV) conditions in an NO_2 environment (p ~10^{-12} Atm, T = 298 K) and found that it's gas-phase presence is negligible. For the chemical equation,

$$2\,\mathrm{NO_2}\,(\mathrm{g}) \leftrightarrow \mathrm{N_2O_4}(\mathrm{g}),$$

The equilibrium constant (K^{eq}) can be expressed as,

$$\mathrm{K^{eq}} = \frac{\mathrm{p^{N_2O_4}}}{[\mathrm{p^{NO_2}}]^2} = \mathrm{e}^{-\frac{\Delta G}{k_B T}},$$

Here, $p^{N_2O_4}$ is the partial pressure of N_2O_4, p^{NO_2} is the partial pressure of NO_2, k_B is Boltzmann's constant, T is temperature, ΔG is change in Gibbs free energy for the gas phase NO_2 dimerization reaction to form N_2O_4. From this equation, the N_2O_4 pressure at equilibrium with NO_2 can be expressed as,

$$\mathrm{p^{N_2O_4}} = [\mathrm{p^{NO_2}}]^2\,\mathrm{e}^{-\frac{\Delta G}{k_B T}},$$

Having an expression for the pressure of N_2O_4 in equilibrium with NO_2 allows us to analyze the concentration of N_2O_4/NO_2 as a function of change in Gibbs free energy at UHV conditions ($p^{NO_2} = 10^{-12}$ Atm, T = 298 K). The result of this analysis is shown in Figure S11. We find that N_2O_4 concentration is close to zero for most of the energy range considered (−0.8 eV to 0.2 eV). It is important to note that ΔG for NO_2 dimerization to N_2O_4 was reported in Ref.[73] to be −0.05 eV at 298 K. The Gibbs free energy range was chosen to favor N_2O_4 formation, yet we find its concentration remains negligible. That the concentration of N_2O_4 is practically zero at relevant changes in Gibbs Free energy means N_2O_4(g) effectively does not exist in equilibrium with NO_2(g) under UHV conditions, and the reaction energies must be considered from the NO_2 gas-phase reference.

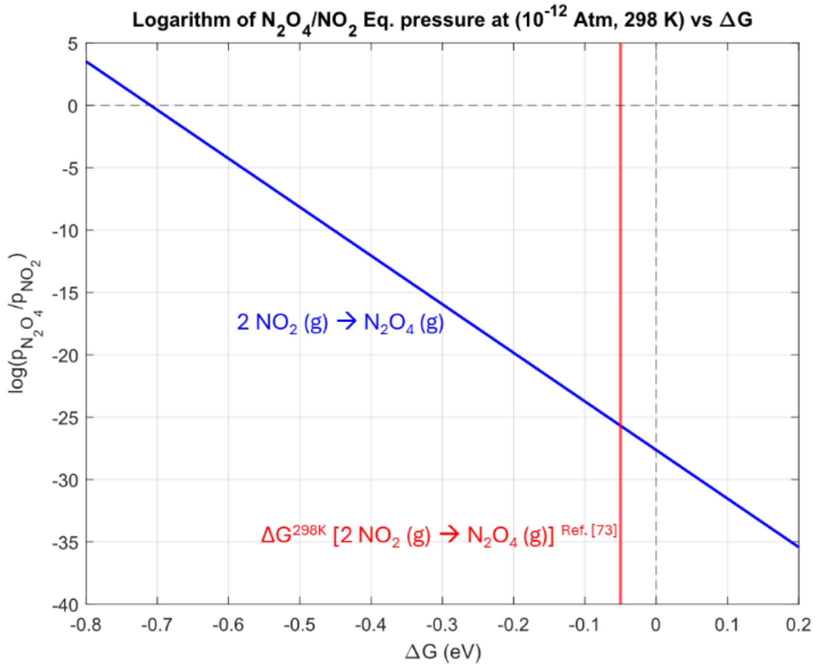

Figure S11: Gas-phase equilibrium concentration of N_2O_4 in an NO_2 environment at p = 10^{-12} Atm. and T = 298 K as a function of Gibbs free energy change for the gas phase NO2 dimerization reaction. Vertical red line shows calculated ΔG for dimerization of NO_2 to N_2O_4 reported in Ref. [73].

Accordingly, to assess the N_2O_4 presence on the silver surface, we then calculated its equilibrium coverage on the surface of the p(4×4) from the reaction's Gibbs free energy, calculating the reaction energy with NO_2(g) as reference, and the expression for Langmuir isotherm coverage,

$$\theta_{N_2O_4} = \frac{K^{eq}p^{rx}}{1 + K^{eq}p^{rx}},$$

Here, K^{eq} is the equilibrium constant for 2 NO_2(g) → N_2O_4* and p^{rx} is the pressure at which the reaction takes place inside the XPS chamber ($p^{rx} = 10^{-12}$ Atm). To calculate the equilibrium constant ($K^{eq} = e^{-\frac{\Delta G}{k_B T}}$) we approximate the change in Gibbs free energy from the equation derived in the section '*Approximation of Gibbs free energy change at 298 K*' (see below) as,

$$\Delta G \approx \Delta E_{rx} - k_B T\, Ln\left(\frac{p^{rx}}{p^0}\right) + \frac{1}{3} T * S_{N_2O_4}{}^{3D-gas}(298.15, p^0)$$

Here, ΔE_{rx} is the reaction energy for 2 NO_2(g)→ N_2O_4*, and has a value of $\Delta E_{rx} = -1.65$ eV, p is the pressure at high vacuum conditions ($p^{rx} = 10^{-12}$ Atm), p^0 is the standard pressure, and

$S_{N_2O_4}{}^{3D-gas}(298.15, p^0)$ is the entropy contribution of the adsorbate, approximated as 1/3 of the 3D gas-phase entropy of N_2O_4 at standard conditions. Taking the standard entropy from NIST, at 298 K this is, $\frac{1}{3}\,\mathrm{T} * \mathrm{S}_{\mathrm{N_2O_4}}{}^{\mathrm{3D-gas}}(298.15, \mathrm{p}^0) = 0.31$ eV. The resulting N_2O_4 surface coverage ($\theta_{\mathrm{N_2O_4}}$) as a function of pressure is presented in Figure S12. We observe from this figure that at p $=10^{-12}$ Atm, the coverage is around 0.02 ML. For comparison the reaction from the inaccessible N_2O_4(g), Erx = −0.72 eV is presented in blue.

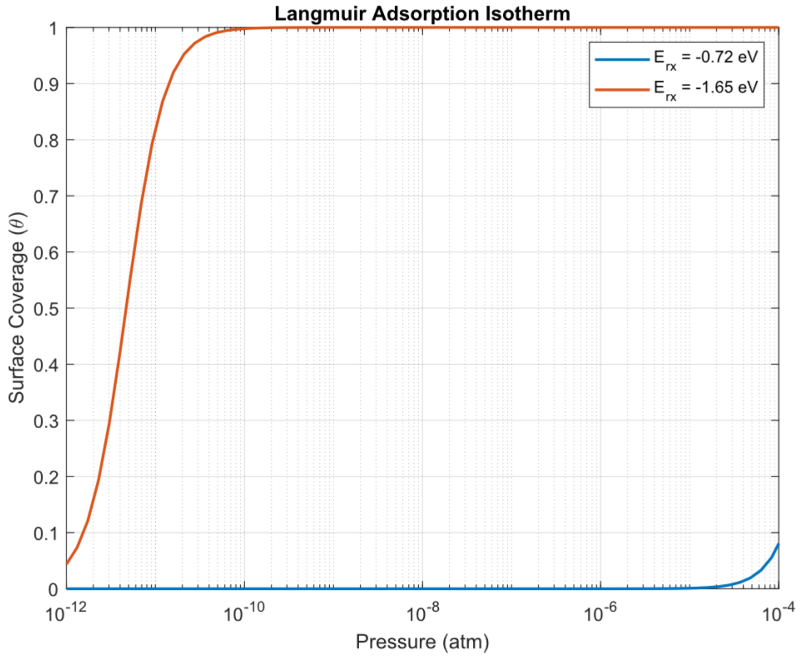

Figure S12: N_2O_4 surface coverage as a function of pressure for different NO_2 dimerization reaction energies. Pressure of 10^{-12} Atm and temperature of 298 K is used in the calculation. Orange line shows the surface coverage of N_2O_4.

Notably, increasing N_2O_4 surface coverage leads to more exothermic reaction energies, further stabilizing the dimers ($[NO_2]_2$). In fact, the reaction energy for $4NO_2(g) \rightarrow 2N_2O_4^*$ is calculated to be −3.12 eV, whereas a value of −4.76 eV is calculated for the adsorption of $6NO_2(g) \rightarrow 3N_2O_4^*$. Importantly, we calculate the dimer differential binding energy per NO_2 to be −0.82 eV and −0.83 eV for the $2N_2O_4$ and $3N_2O_4$ configurations, respectively. In comparison, we calculate the differential binding energy for 2 and 3 adsorbed NO_2 molecules to be −1.21 eV and −0.75 eV, respectively.

To evaluate the thermodynamically preferred adsorption configuration on the p(4×4), we calculated the surface energy change as a function of NO_2 chemical potential with the use of *ab-initio*

thermodynamics and the NIST thermochemistry tables.[S15] Given that the adsorbates are of non-negligible size, we account for the entropy of the adsorbate on the surface as part of the expression for the surface energy,

$$\Delta\gamma(T,p) = \frac{1}{A}\left[E_{ads/\mathrm{p}(4\times4)} - E_{\mathrm{p}(4\times4)} - N_{NO_2}\mu_{NO_2}(T,p) - N_{NO_2}\,TS^{ads}(T,p)\right]$$

As done in the main manuscript, A is the area of the p(4×4) cell, whereas T is temperature set to 298 K. N_{NO_2} is the number of NO_2 molecules in the surface unit cell. $E_{ads/\mathrm{p}(4\times4)}$ is the energy of the entire slab/adsorbate system, while $E_{\mathrm{p}(4\times4)}$ is the energy of the p(4×4) slab without adsorbates. Moreover, $\mu_{NO_2}(T, p)$ is the chemical potential of NO_2 at the respective temperature and pressure. Finally, $S^{ads}(T,p)$ is the entropy of the adsorbate, approximated as 1/3 the entropy of the NO_2 gas-phase species. The results of this analysis are presented in Figure S13. From the thermodynamic analysis, we find that $3[NO_2]_2$ (which is 3 N_2O_4) surface coverage is thermodynamically preferred over lower monomer NO_2 coverages at UHV conditions.

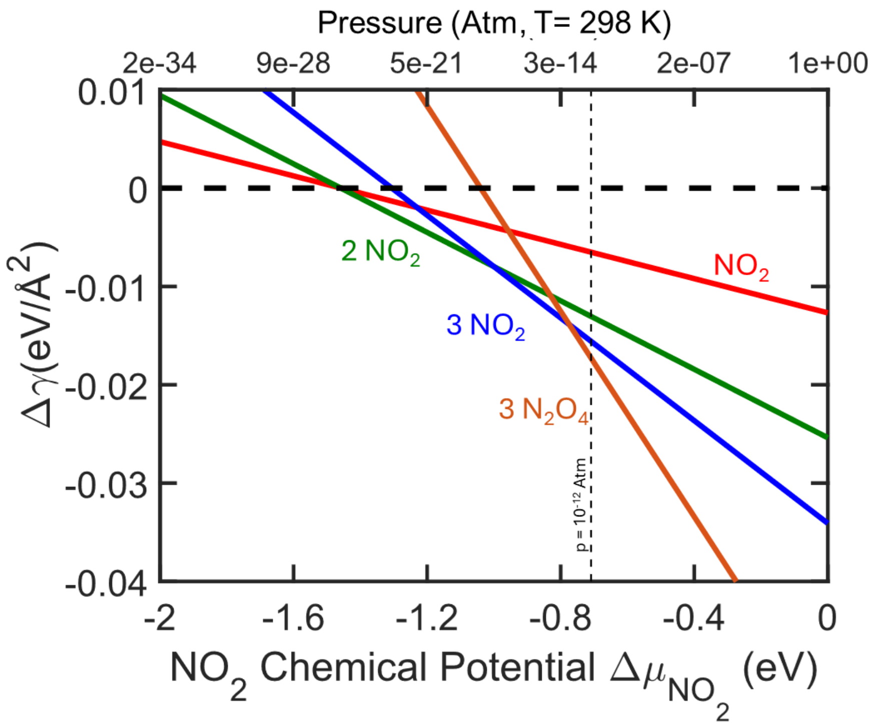

Figure S13: Surface stability upon NO_2 adsorption for different surface coverages on p(4×4)-Ag(111) as a function of nitrogen dioxide chemical potential ($\Delta\mu_{NO_2}$). The corresponding pressure is plotted on the top axis at T = 298 K. The pristine surface (with no adsorbates) is shown as a dashed line at zero Δγ energy. The chemical potential equivalent to a pressure of 10^{-12} Atm at 298 K is shown as a dashed vertical line.

Approximation of Gibbs free energy change at 298 K.

We calculate the change in Gibbs free energy as,

$$\Delta \mathbf{G} = \Delta \mathbf{H} - \Delta \boldsymbol{\mu} - \mathbf{T} \cdot \mathbf{S}^{\mathbf{2D-gas}}(\mathbf{T}, \mathbf{p^0})$$

Here, $\mathbf{\Delta H}$ is the change in energy upon adsorption, calculated as the adsorption energy of the N_2O_4 on the p(4x4) surface. $\boldsymbol{\Delta\mu}$ is the molecular chemical potential, and $\mathbf{S^{2D-gas}}(\boldsymbol{T}, \boldsymbol{p^0})$ is the entropy of a 2-dimensional ideal gas at standard pressure and temperature T. We express the chemical potential the gas-phase species, $\boldsymbol{\Delta\mu}$, as,

$$\Delta \boldsymbol{\mu} = \Delta \boldsymbol{\mu}_{\mathbf{0}} + \mathbf{k_B T Ln}\left(\frac{\mathbf{p}}{\mathbf{p^0}}\right)$$

Here $\boldsymbol{\Delta\mu_0}$ is the standard chemical potential, while the logarithmic term accounts for the entropic contribution caused by the change in pressure with respect to the standard state. The standard chemical potential is expressed as,

$$\Delta \boldsymbol{\mu}_{\mathbf{0}} = [\mathbf{H}(\mathbf{T}; \mathbf{p^0}) - \mathbf{H}(\mathbf{298.15\ K};\ \mathbf{p^0})] - \mathbf{T} \cdot \mathbf{S}(\mathbf{298.15\ K};\ \mathbf{p^0})$$

At 298 K, the enthalpy difference becomes effectively zero, while entropy at the standard state of a 3-D gas can be calculated from NIST thermodynamic tables.[S15] Hence, we can rewrite the standard chemical potential as,

$$\Delta \boldsymbol{\mu}_{\mathbf{0}} = -\mathbf{T} \cdot \mathbf{S}(\mathbf{298.15\ K};\ \mathbf{p^0}) = -\mathbf{T} \cdot \boldsymbol{S}^{\boldsymbol{3D-gas}}(\mathbf{298.15}\ \boldsymbol{K}, \boldsymbol{p^0})$$

Now we rewrite the Gibbs free energy expression as,

$$\Delta \mathbf{G} = \Delta \mathbf{E} - \mathbf{k_B T Ln}\left(\frac{\mathbf{p}}{\mathbf{p^0}}\right) + \mathbf{T} \cdot \mathbf{S}^{\mathbf{3D-gas}}(\mathbf{298.15\ K}, \mathbf{p^0}) - \mathbf{T} \cdot \mathbf{S}^{\mathbf{2D-gas}}(\mathbf{T}, \mathbf{p^0})$$

We can grossly approximate the entropy difference between a S^{3D}-S^{2D} as 1/3 of the 3-D gas entropy. Since we assume the one-dimensional loss affects equally the translational, rotational, and vibrational

degrees of freedom we effectively slightly overestimate calculated entropy difference. Considering the 2D gas phase entropy at standard conditions (T = 298.15 K) we get,

$$\Delta G \approx \Delta H - k_B T \mathrm{Ln}\left(\frac{p}{p^0}\right) + \frac{1}{3} S^{3D-gas}(298.15\ K, p^0)$$

Core-level shifts: Assumptions and Limitations

Accurate determination of core-electron binding energies from *ab initio* calculations is not trivial. On one hand, absolute core-electron binding energies (BE) from *ab initio* calculations can be difficult to ascertain, as they require a very accurate description of the electronic structure. Moreover, calculation of the core-electron BE from the calculated eigenvalues, as done when calculating absolute core-electron BE's, neglect the screening effect of the electronic relaxation of the valence electrons upon core-electron emission, which is known to affect the experimentally measured core-electron binding energy.[S16] On the other hand, relative core-levels shifts (CLS), as the ones performed in this work, capitalize on cancellation of errors but do not provide an absolute value for the binding energy, making comparison to experimental measurements difficult.

In this work, we used the double-reference method which allows for accurate CLS calculations that can be easily converted for comparison with experimental measurements.[S17] In the CLS calculations performed here, we have exploited cancelation of errors by having the reference system embedded within the overall system of interest. Specifically, our reference system consists of an N or O atom embedded in an interstitial site in the bulk-constrained layers. Accordingly, having a well-defined experimental BE for N 1s (NO/p(4×4)) and O 1s (oxygen on the p(4×4)) reference permits easy connection between the calculated relative energies to the absolute values measured experimentally. The present methodology is, however, not without limitations. Relative CLS calculations are known to be highly sensitive to the choice of exchange correlation functional.[S18] Core-electron binding energies can be understood as a result of the electrostatic interaction of the core-electron with its surrounding media. Hence, having an appropriate description of the electronic structure of the system of interest is necessary to capture accurate relative changes in binding energies. In fact, gas phase CLS calculations for molecules are well known to require hybrid functionals, which localize charge to a much larger degree than GGA functionals, in order for the CLS to be comparable to experimental measurements.[S18] Similarly,

for strongly correlated extended systems, the use of hybrid functionals tend to give a more accurate value of the calculated shifts.[S18,S19] Importantly, systems where the electronic structure is delocalized, like metallic systems, are generally well described with GGA functionals.[S20,S21] Hence, the choice of exchange-correlation functional must be careful. Additional limitations, like poor description of the electronic structure of the adsorbates or choice of charge-compensation method upon creation of the core-hole are a prevalent and unavoidable issue that can only be moderately mitigated with systematic evaluation. Ultimately, to have certainty that the relative CLS calculated values are meaningful, different surface conditions and different adsorbates should be systematically evaluated. Finally, comparison to experimental measurements is essential to determine the accuracy of CLS calculations. The methodology used here has been extensively utilized and it is known to provide accurate CLS. [S3,S5,S19,S21,S22]

References.

S1. Yu, H., Szilvási, T., Wang, K., Gold, J. I., Bao, N., Twieg, R. J., Mavrikakis, M., Abbott, N. L., Amplification of elementary surface reaction steps on transition metal surfaces using liquid crystals: dissociative adsorption and dehydrogenation. *J. Am. Chem. Soc.*, **2019**, 141(40), 16003-16013.

S2. Hammer, B., Nørskov, J. K., Electronic Factors Determining the Reactivity of Metal Surfaces., **1995**, *Surf. Sci.*, 343(3), 211–220.

S3. Tal, A. A., Posada-Borbón, A., Grönbeck, H. J., Abrikosov, I. A., Correlation between Ethylene Adsorption Energies and Core-Level Shifts for Pd Nanoclusters., *J. Phys. Chem. C*, **2019**, 123, 2544−2548.

S4. Kandoi, S., Ferrin, P.A., Mavrikakis, M., Hydrogen on and in Selected Overlayer Near-Surface Alloys and the Effect of Subsurface Hydrogen on the Reactivity of Alloy Surfaces., *Top. Catal.*, **2010**, 53, 384–398.

S5. Posada-Borbón, A.; Grönbeck, H. Hydrogen adsorption on $In_2O_3(111)$ and $In_2O_3(110)$. *Phys. Chem. Chem. Phys.* **2020**, 22, 16193–16202.

S6. Frey, B. L., Corn, R. M., Weibel, S. C., "Polarization-modulation approaches to reflection-absorption spectroscopy," in Handbook of Vibrational Spectroscopy (John Wiley & Sons, Ltd., 2006), pp. 1042–1056.

S7. Cadwell K.D., Alf M.E., Abbott N.L., Infrared spectroscopy of competitive interactions

between liquid crystals, metal salts, and dimethyl methylphosphonate at surfaces. *J. Phys. Chem. B.,* **2006,** 10(51), 26081-8.

S8. Blaylock, D. W.; Ogura, T.; Green, W. H.; Beran, G. J. O. Computational investigation of thermochemistry and kinetics of steam methane reforming on Ni(111) under realistic conditions. *J. Phys. Chem. C*, **2009**, 113, 4898–4908.

S9. Peterson, A. A.; Abild-Pedersen, F.; Studt, F.; Rossmeisl, J.; Nørskov, J. K. How copper catalyzes the electroreduction of carbon dioxide into hydrocarbon fuels. *Energy Environ. Sci.* **2010**, 3, 1311–1315.

S10. Granda-Marulanda, L. P.; Rendón-Calle, A.; Builes, S.; Illas, F.; Koper, M. T. M.; Calle-Vallejo, F. A Semiempirical Method to Detect and Correct DFT-Based Gas-Phase Errors and Its Application in Electrocatalysis. *ACS Catal.* **2020**, 10, 6900–6907.

S11. Studt, F.; Abild-Pedersen, F.; Varley, J. B.; Nørskov, J. K. CO and CO_2 Hydrogenation to Methanol Calculated Using the BEEF-vdW Functional. *Cat. Lett.* **2013**, 143, 71–73.

S12. Studt, F.; Behrens, M.; Kunkes, E. L.; Thomas, N.; Zander, S.; Tarasov, A.; Schumann, J.; Frei, E.; Varley, J. B.; Abild-Pedersen, F.; Nørskov, J. K.; Schloegl, R. The mechanism of CO and CO_2 hydrogenation to methanol over Cu-based catalysts. *ChemCatChem* **2015**, 7, 1105–1111.

S13. Posada-Borbón, A.; Grönbeck, H. A First-Principles-Based Microkinetic Study of CO_2 Reduction to CH_3OH over $In_2O_3(110)$. *ACS Catal*. **2021**, 11, 9996–10006.

S14. Urrego-Ortiz, R.; Builes, S.; Calle-Vallejo, F. Automated versus Chemically Intuitive Deconvolution of Density Functional Theory (DFT)-Based Gas-Phase Errors in Nitrogen Compounds. *Industrial & Engineering Chemistry Research* **2022**, 61, 13375–13382.

S15. Chase, M.W., Jr., *NIST-JANAF Themochemical Tables, Fourth Edition*, J. Phys. Chem. Ref. Data, Monograph 9, 1998, 1-1951. [all data]. NO_2 data retrieved online from https://webbook.nist.gov/cgi/cbook.cgi?ID=C10102440&Mask=1#Thermo-Gas [Accessed: 2025-08-25]. N_2O_4 data retrieved online from https://webbook.nist.gov/cgi/cbook.cgi?ID=C10544726&Mask=1#Thermo-Gas [Accessed: 2023-05-16].

S16. Pueyo Bellafont, N.; Bagus, P.S. ; Illas, F. Prediction of core level binding energies in density functional theory: Rigorous definition of initial and final state contributions and implications on the physical meaning of Kohn-Sham energies. *J. Chem. Phys.* **2015**, 142, 214102.

S17. Lizzit, S.; Baraldi, A.; Groso, A.; Reuter, K.; Ganduglia-Pirovano, M. V.; Stampfl, C.;

Scheffler, M.; Stichler, M.; Keller, C.; Wurth, W.; Menzel, D. Surface core-level shifts of clean and oxygen-covered Ru(0001). *Phys. Rev. B,* **2001**, 63 (20), 205419

S18. Van den Bossche, M. ; Martin, N. M.; Gustafson, J. ; Hakanoglu, C. ; Weaver, J. F.; Lundgren, E.; Grönbeck, H., Effects of non-local exchange on core level shifts for gas-phase and adsorbed molecules. *J. Chem. Phys.*, **2014**, 141, 034706.

S19. Gericke, S. M. ; Kauppinen, M.M. ; Wagner, M. ; Riva, M. ; Franceschi, G. ; Posada-Borbón, A. ; Rämisch, L. ; Pfaff, S. ; Rheinfrank, E. ; Imre, A. M. ; Preobrajenski, A. B. ; Appelfeller, S. ; Blomberg, S. ; Merte, L. R. ; Zetterberg, J. ; Diebold, U. ; Grönbeck, H. ; Lundgren, E., *ACS Appl. Mater. Interfaces*, **2023**, 15, 45367-45377.

S20. Nilsson, V. ; Van den Bossche, M. ; Hellman, A. ; Grönbeck, H., Trends in adsorbate induced core level shifts, *Surf. Sci.*, **2015**, 640, 59-64.

S21. Posada-Borbón, A. ; Hagman, B. ; Schaefer, A. ; Zhang, C. ; Shipilin, M.; Hellman, A. ; Gustafson, J. ; Grönbeck, H., Initial oxidation of Cu(100) studied by X-ray photo-electron spectroscopy and density functional theory calculations, *Surf. Sci.*, **2018**, 675, 64-69.

S22. Posada-Borbón, A. ; Bosio, N. ; Grönbeck, H., On the signatures of oxygen vacancies in O1s core level shifts, *Surf. Sci*., **2021**, 705, 12176.
